# Skill-Mix Changes Targeting Health Promotion and Prevention Interventions and Effects on Outcomes in all Settings (Except Hospitals): Overview of Reviews

**DOI:** 10.3389/ijph.2023.1605448

**Published:** 2023-05-09

**Authors:** Claudia Bettina Maier, Juliane Winkelmann, Laura Pfirter, Gemma A. Williams

**Affiliations:** ^1^ Department of Health Care Management, Faculty of Economics and Management, Technical University Berlin, Berlin, Germany; ^2^ European Observatory on Health Systems and Policies, Brussels, Belgium; ^3^ European Observatory on Health Systems and Policies, London School of Economics and Political Science, London, United Kingdom

**Keywords:** health promotion, prevention, outcomes, skill-mix, task-shifting, outreach, role expansion

## Abstract

**Objectives:** Skill-mix changes to step up health promotion and prevention are increasing, but there is limited evidence on their effects.

**Methods:** Overview of reviews, based on a protocol. The search was carried out in six databases, screening was performed ensuring high interrater reliability. All countries, health professions and lay workers in all settings (except hospitals) were included, quality appraisals performed.

**Results:** A total of 31 systematic reviews were included. Expanded roles performing outreach (e.g., home visits) had mostly positive effects on access and health outcomes, primarily for hard-to-reach groups. Task-shifting in colorectal or skin cancer screenings (performed by advanced practice nurses) were suggested effective; supporting roles (by community health workers) increased uptake in screenings, but based on limited evidence. Expanded roles of various professions focusing on lifestyle modification showed promising effects in most reviews, including weight, diet, smoking cessation and physical activity. Reviews on cost-effectiveness were based on limited evidence.

**Conclusion:** Promising skill-mix changes included expanded roles providing lifestyle modifying interventions, task-shifting, and outreach roles for hard-to-reach groups, whereas evidence on costs was limited.

## Introduction

The rise in chronic conditions globally (1–3) has increased policy attention on health promotion, reinforced by the 2018 Astana declaration (4, 5). Developing healthy policies and integrating health promotion in primary care have shown some, albeit limited progress (6, 7). Primary care services focus on curative care and less on prevention (8), while health professionals often face high workloads and lack the necessary skills (9). In response, several countries have changed the skill-mix of their workforce, defined as changes to the skills, roles or tasks of individual health professions or teams (10, 11). Examples are new roles (e.g., outreach), task-shifting or multiprofessional collaboration (10–12). In Europe, the health workforce has increasingly diversified (13), in the United States (U.S.), high-performing primary care practices have included new professional roles, outreach and patient coaching (14).

Frequent skill-mix changes have been introduced among nurses and pharmacists. An OECD study highlighted that in many countries, nurses work in expanded roles in prevention (15). The roles of pharmacists increasingly include prevention activities, in particular since the COVID-19 pandemic (16, 17). Other studies covered physician assistants, medical assistants or dental hygienists (18–20). Moreover, community health workers (CHW) are increasingly working in prevention (21–23). Reasons triggering skill-mix changes have been referred to as provider shortages, limited access to services, increasing chronic conditions and the need for tailored services for hard-to-reach populations (11, 24, 25).

Several systematic reviews exist on skill-mix, but often focus only on single professions or on a narrow set of outcomes. This paper takes a larger study on skill-mix as a starting point (24), which consisted of an overview of reviews and country case studies, published as a policy-focused book (25). It found that many European countries have adopted strategies on health promotion and patient-centred care and identified a range of reviews on skill-mix across the life cycle (25). However, the evidence was synthesized at a highly aggregate level, a detailed analysis of individual reviews was not undertaken, it focused on Europe and other high-income countries and the search was conducted in 2018.

The present study therefore aimed to: i) update the identification of systematic reviews on skill-mix in health promotion, primary or secondary prevention; ii) cover all countries; iii) apply a skill-mix typology; iv) synthesise the evidence on outcomes for groups of interventions.

## Methods

This overview of systematic reviews (26, 27) expands and updates the evidence of a previous study on skill-mix innovation, effectiveness and implementation (24, 25). The protocol was registered in PROSPERO (Nr. CRD42018090272) (28), published in March 2018, when the search had taken place and screening process had started, but was not finalised. For this study, there were the following deviations from the protocol. First, for this paper, there was no year restriction in the search applied, contrary to the protocol and earlier work which covered the years 2010–2018 only (25). This study provided an update of the search in June 2021. Second, this paper focused on health promotion, primary and secondary prevention; whereas the protocol and previous work covered more areas (e.g., health promotion, prevention, rehabilitation, management of diseases) (24, 25). Third, high, middle- and low-income countries were included, contrary to the protocol and earlier work (25) which focused on Europe and other high-income countries and excluded reviews that only covered low- and middle-income countries. Finally, this study applied skill-mix typologies, which was not performed in earlier work (24, 25), and used three typologies (instead of four as originally specified in the protocol, see section: data synthesis).

### Definition

Skill-mix changes were defined as new or changing roles, tasks or skills of health professionals and/or teams (28). Examples include role expansions (e.g., new roles, roles which did not exist previously or were not routinely performed); task-shifting (from higher to lower qualified professions); and team work (multiprofessional collaboration) (10, 24, 29). All health professions were included (e.g., physicians, nurses, midwives, physician assistants, pharmacists, medical assistants) as well as CHWs with no or limited training.

### Types of Studies

All systematic reviews with narrative or meta analysis were included with comparison groups on skill-mix changes and outcomes. Excluded were rapid reviews, scoping reviews, integrative reviews, protocols and other studies. Reviews published in languages other than English were excluded.

### Population and Setting

All population groups and individuals in ambulatory care, community or other settings, including at home were included. Interventions that focused on the interface between inpatient and ambulatory care were also included. Hospitals were excluded, as was emergency medicine.

### Types of Intervention

Interventions covered skill-mix changes providing health promotion (for healthy populations) or prevention activities (preventing the onset of diseases). Examples include prevention for population group (e.g., maternal and child health (MCH), immunization, screenings) or individual at-risk factors (e.g., smoking, weight/obesity) (4, 30). This review covered primary and secondary prevention, but excluded tertiary prevention. The latter is commonly addressed by improvements of treatment and medication regimens or rehabilitation, which was not the focus of this study (4, 30) contrary to earlier work (25).

### Comparison

The comparison groups were either standard-of-care, usual care or previous skill-mix configurations as identified in the reviews.

### Outcome Measures

Primary outcomes were individual or population-related outcomes (e.g., health- or disease-specific, mortality, patient experience) and health system related outcomes (e.g., access to services, resource use, costs).

### Search Method

#### Electronic Searches

The search strategy was reviewed internally, piloted, and developed with a librarian specialised in conducting systematic searches. It was conducted in January 2018 and updated on 24 June 2021. It was run in Embase first, then adapted to the following databases: Medline in Ovid, Cochrane CENTRAL, Web of Science Core Collection, CINAHL EBSCOhost (Cumulative Index to Nursing and Allied Health Literature), PsychINFO Ovid and Google scholar. Search terms included combined Medical Subject Headings with free text words (Supplementary Material S1). Filters were used depending on the databases to identify systematic reviews. No supplementary primary studies were included. In addition to the electronic search, snowballing was used to identify other reviews by going through the reference lists of relevant reviews.

### Data Collection and Analysis

#### Selection of Reviews

**FIGURE 1:**
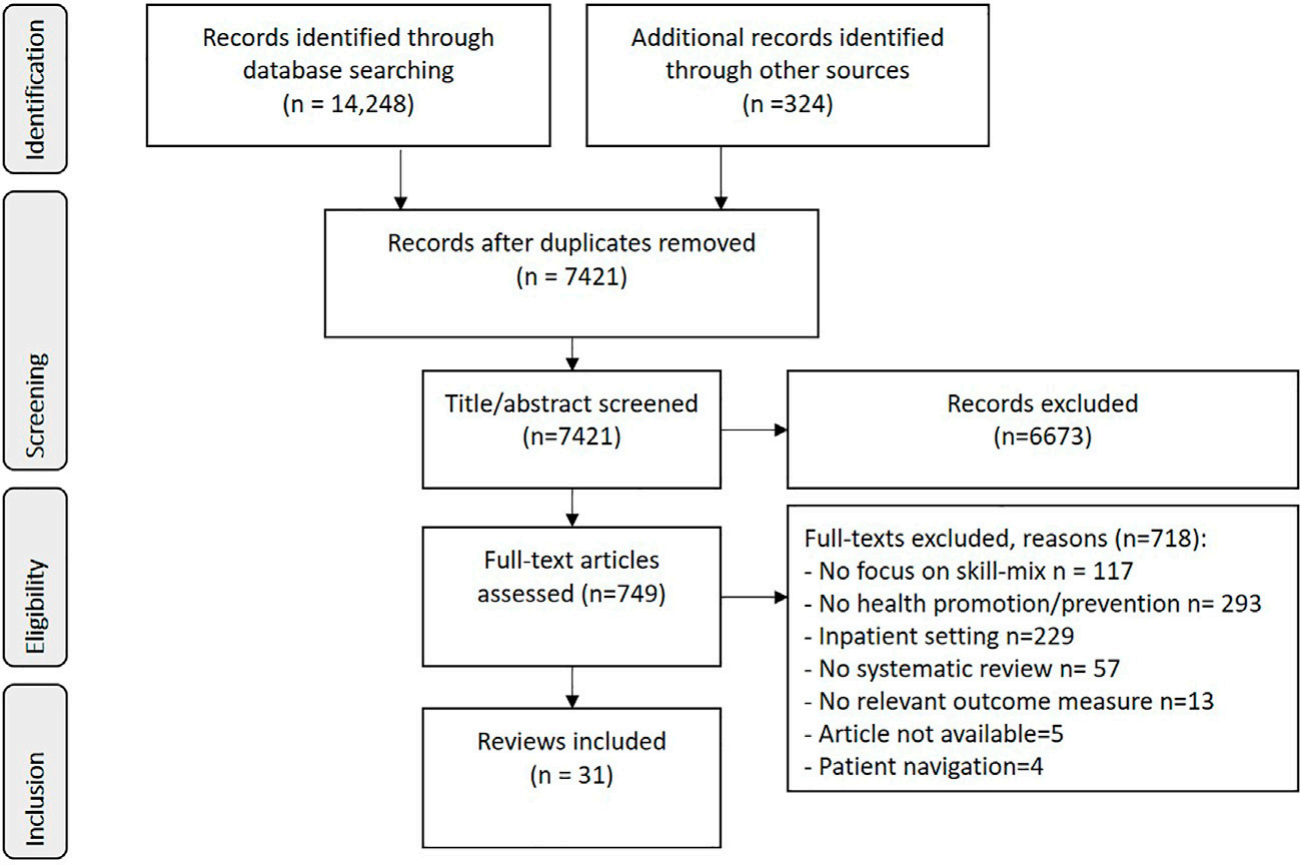
Search and article selection (2021, all countries included).

The electronic search produced a total of 14,572 hits from electronic databases and snowballing (see PRISMA Flow Diagram (31), Figure 1). After the removal of duplicates, screening of the titles and abstracts was executed by CM, JW, MK in Rayyan (32). Prior to the screening, the title/abstracts of the first 100 hits and the first 20 full-text articles were screened by the three researchers resulting in high agreement rates (title/abstract: 0.86, full-text: 0.78), using an extended version of Cohen’s kappa coefficient which is suitable for three researchers (33, 34). Of the 749 full-text versions assessed by the researchers, 718 were excluded (see Figure 1).

#### Data Extraction

An excel data extraction form was used (35), covering country, participants, professions, intervention, comparison group, care settings and outcomes. A piloting phase was performed, during which one researcher double-checked the data extraction of the first five reviews of the other reviewers. Differences were resolved through discussion.

#### Data Synthesis

Data was narratively synthesized. Meta-analysis was not possible due to the heterogeneity of the interventions and outcomes. Data synthesis was undertaken for the following themes: MCH; screenings; vaccination; lifestyle modification related to Smoking, Nutrition, Physical Activity, Weight reduction/management (SNAPW) (36). Potential overlap in individual studies, e.g., the population, interventions, comparators, and/or outcomes was not examined.

The interventions described in the reviews were categorized into the following three skill-mix typologies: 1) task-shifting, defined as shifting tasks from a higher to a lower qualified professional/worker (or as part of teams) with a higher qualified professional in the comparison group; 2) role expansion, defined as new roles, professions which did not exist previously or were not routinely performed, with various comparison groups (other professions, usual care), 3) other/hybrid/various, defined as all other interventions. In the protocol, it was planned to also use additional typologies on team work and collaboration, but this was not feasible, hence a third category was used instead. Reviews were grouped by typology, cadres (by health professions, lay workers), outcomes and population groups. All skill-mix changes were covered, except for the role of patient navigators, which was covered elsewhere (37).

We followed the Preferred Reporting Items for Overview of Reviews (PRIOR) guideline developed for overview of reviews of healthcare interventions (Supplementary Material S2) (38).

#### Quality Assessment

Quality appraisals were performed two researchers (LP, CM), after piloting, using the Assessment of Multiple Systematic Reviews (AMSTAR) II checklist (39). Inconsistencies were discussed until agreement was reached. Summary scores for each review and by sub-themes were provided.

## Results

A total of 31 reviews were included. Seven reviews focused on skill-mix changes on MCH, five on screenings, two on vaccinations and 18 on lifestyle modifications (Table 1). One review covered both vaccination and MCH (22). Eight reviews focused on changes to the roles of nurses (and midwives), and four on pharmacists and lay workers/community health workers. In terms of skill-mix typology, 15 reviews analysed role expansions, four task-shifting from higher to lower qualified professions or teams. Among the remaining 12 reviews, either task-shifting and role expansion were both covered, hybrid or multiple models were analysed (e.g., case manager roles and introducing team work), or it was unclear what skill-mix typology was covered.

**TABLE 1:** Overview of the systematic reviews (various countries, search performed in 2021).

Reviews by prevention area	Reviews		Profession					Skill-mix typology		
	Nr. of reviews	ME	Nurse (& midwife)	Pharmacist	CHW/lay worker	Other	Various	Role expansion	Task shifting	Other/various/hybrid models
Maternal and Child health (MCH)	7*	1*	1	1	1*	0	5	4	1	2*
Screenings	5	1	2	0	2	0	1	2	2	1
Vaccinations	2*	1*	0	1	1*	0	0	0	0	2*
Lifestyle modification (SNAPW)	18	7	5	2	1	4	6	9	1	8
TOTAL	31*	9*	8	4	4*	4	12	15	4	12*

Notes: * one review (22) performed meta-analyses with outcomes on both, MCH and vaccinations, hence is listed under “MCH” and “Vaccinations” but listed only once under TOTAL; CHWs, Community Health Workers; MCH, Maternal and Child Health; ME, Meta Analysis; SNAPW, Smoking, Nutrition, Physical Activity, Weight reduction/management.

### Quality of the Reviews

The quality appraisal ranged from low to high quality (summary scores: 7 to 29 points) (Table 2). The lowest mean score was identified for the reviews on screening, the highest on lifestyle modification. A full list of the AMSTAR II appraisals is available in Supplementary Material S3.

**TABLE 2:** Summary of quality appraisals (various countries, search performed in 2021).

Areas covered	Nr of reviews appraised	Range of sum scores per review	AMSTAR II mean score
Maternal and Child Health (MCH)	7*	8–24	14.86*
Screening	5	7–19	11.40
Vaccinations	2*	10, 24	17.00*
Lifestyle (SNAPW)	18	7–29	17.89
TOTAL	31*	7–29	16.16*

Notes: *one review (22) performed meta-analyses with outcomes on both, MCH and vaccinations, hence is listed under “MCH” and “Vaccinations” but listed only once under TOTAL. For the scoring, however, the review was included twice in the total sum score and sub-group analyses. AMSTAR II (39): Assessment of Multiple Systematic Reviews, MCH: Maternal and Child Health, SNAPW: Smoking, Nutrition, Physical Activity, Weight reduction/management.

### Maternal and Child Health (MCH)

Of the seven reviews (Table 3), one analysed task-shifting, whereby midwives or midwife/GP teams took over routine visits of women with low-risk pregnancy from obstetricians/gynecologists (40). Four reviews focused on role expansions, of which one analysed pharmacists providing emergency contraception (41) and three prenatal and/or postnatal home visits delivered by nurses, CHWs or multiprofessional teams (42–44). The remaining two reviews covered multiple skill-mix typologies, one subsumed task-shifting and role expansion performed by lay workers (22), the other changes to team work and introducing case manager roles (45).

**TABLE 3:** Skill-mix and maternal and child health (various countries, search performed in 2021).

Skill-mix typology	Description of Intervention	Profession in intervention	Profession in comparison	Nr studies	ME	Setting	Country	Population	Outcomes	Source
TS	Routine management of low-risk pregnancy	Midwife/GP-managed	Obstetrician/gynaecologist	3	-	Clinics, GP offices, other	High-income countries	1528 low-risk pregnant women in intervention, 1547 in control	• 3 trials (midwife/GP-managed vs. obstetrician/gynaecologist) with similar outcomes: Caesarean section, anaemia, UTI, postpartum haemorrhage	(40)
									• Higher satisfaction with continuity of care in midwife/GP-model	
									• Cost reduction in intervention, evidence limited	
RE	Home visits, aimed at reducing health disparities and addressing social determinants of health	Primarily by nurses or CHWs, other	n/r	39	-	Home/community	US	Native American or other ethnic minority mothers, often teen mothers	• Significantly improved parenting knowledge and self-efficacy skills	(43)
									• Significantly improved parenting behavior, reduced parenting stress, and maternal depression	
									• Significantly fewer overnight hospital stays (p < 0.01)	
RE	Prenatal home visits	Various, including nurses and others (not consistently reported)	n/r	28 (14 RCTs)	-	Home/community	US, not consistently reported	High-risk pregnant women	• Of 24 studies on birth weight, 7 with significant increase (23-64g, one outlier of 405g)	(42)
									• Of 16 studies on gestational age, 4 with significant positive effect, 3 with nonsignificant but positive effect	
									• 8 of 11 studies with improved care utilization, of which five (42%) statistically significant increase in adequate prenatal care use	
RE	Home visits during pregnancy or up to 6 months after birth for the prevention of child maltreatment	Nurses & midwives (n = 11), other, multidisciplinary teams (n = 5)	n/r	33	-	Home/community	US (n = 24), Australia, New Zealand	Pregnant women, women with newborn, often high-risk families	• 7 of 22 programmes (32%) of at least adequate quality were cost saving when including lifetime costs	(44)
									• The most cost-effective programmes used professional home visitors (e.g., nurse, nurse/midwife, social worker) in a team for high risk populations and included multiple interventions	
RE	Pharmacist role in reducing unintended pregnancy (e.g., access to emergency contraception, hormonal contraception and injection)	Pharmacists alone, pharmacist-physician partnerships	n/r	38	-	Community pharmacists	US	Women of reproductive age	• Improvements in access to emergency contraceptive (8 studies)	(41)
									• Up to 700 pregnancies prevented (1 pilot study)	
									• No differences in pregnancy rates, STI infections, sexual risk-taking behaviour, condom use (1 study)	
									• Increased satisfaction for pharmacist-initiated hormonal contraception (1 study)	
O	Teams or case management, often combined with other measures (e.g., patient education, IT, social service referrals, multilingual services)	Various, including nurses, social worker, obstetrician, gynecologist, certified nurse-midwife	n/r	33	-	Primary Care	US	Pregnant women, women with newborn	• Of 13 studies on team work and infant birth outcomes, 54% (N = 7) with significant improvements	(45)
									• Of 24 studies on case management and infant birth weight, 12 (50%) with significant improvements	
									• Of 22 patient education programmes and birth weight, 50% with improved outcomes	
									• Of 3 multilingual programmes and birth weight, 67% with improved outcomes	
O	Lay health workers roles in maternal and child health	Lay health workers (with some training), e.g., CHW, birth attendants, peer counsellors, home visitors	n/r	63	yes	Home visits, primary care, community-based, plus phone calls	AU, CA, NZ, UK, US, IE, BR, IN, MX, TH, ZA, TR, BD, VN, other	Pregnant women, women with newborn	• Initiation of breastfeeding (12 studies) (RR = 1.36, 95% CI 1.14 to 1.61; p < 0.00001), any breastfeeding (12) (RR 1.24, 95% CI 1.10 to 1.39; p = 0.0004) and exclusive breastfeeding (12) (RR 2.78, 95% CI 1.74 to 4.44; p < 0.0001)	(22)
									• LHWs may reduce child morbidity (7) (RR 0.86, 95% CI 0.75 to 0.99; p = 0.03); child mortality (3) (RR 0.75, 95% CI 0.55 to 1.03; p = 0.07) and neonatal mortality (4) (RR 0.76, 95% CI 0.57 to 1.02; p = 0.07)	
									• Care seeking practice: Insignificant increase (3) (RR 1.33, 95% CI 0.86 to 2.05; p = 0.20)	

Notes: one review (22) is listed in this table and Table 4, results were extracted by outcome measures, hence, the review itself is listed twice, but the individual studies are presented only once. Abbreviations: CHW, Community health workers; GP, General Practitioner; LHW, Lay health worker; O, hybrid/various/other; RCT, Randomized Control Trial; RE, role expansion; STI, sexually transmitted infections; TS, task shifting; UTI, Urinary tract infection. Country abbreviations: AU, Australia; CA, Canada; NZ, New Zealand; UK, United Kingdom; IE, Ireland; BR, Brazil; IN, India; MX, Mexico; BD, Bangladesh; TR, Turkey; TH, Thailand; ZA, South Africa; VN, Vietnam; US, United States of America.

The review on task-shifting (40) showed no differences in the majority of clinical outcomes between the midwife/GP and obstetritian/gynecologists model. There was higher satisfaction with continuity of care and indication of reduced costs in the midwife-GP model. The midwife/GP model had lower rates of pregnancy-induced hypertension and pre-eclampsia as well as preterm delivery and antepartum haemorrhage; however, recognition of fetal malpresentation tended to be higher in the obstetritian/gynecologist group.

In the three reviews analysing pre- or postnatal home visits, most interventions were delivered by nurses, CHWs or teams and covered parenting skills improvements, lactation support, education or substance abuse prevention focusing on ethnic minorities or at-risk groups. Significantly improved outcomes were shown for increased parenting knowledge, reduced stress, maternal depression and fewer hospital stays, if targeted at ethnic minorities or focused on addressing social determinants of health (43). Improved utilization of prenatal care were found (8 of 11 studies), but mixed results on birth weight and gestational age (42). The economic analysis (44) showed large variation in the number of home visits per programme and the educational background of the home visitors. It found variation in the incremental costs of home visits, ranging from USD 1,800 to 30,000 per family, and in the estimated cost-effectiveness per case of maltreatment prevented.

The review analysing pharmacists with expanded roles in providing emergency contraception (e.g., provision of depot reinjection, initiation of oral contraceptives) (41) found improvements for women in accessing emergency contraceptives. There was limited evidence on prevention of pregnancies, increased patient satisfaction and several non-significant outcomes were reported (e.g., pregnancy rates) (ibid.). The two remaining reviews covering multiple skill-mix typologies including teamwork and lay health workers providing MCH services (22, 45), showed statistically improved outcomes on breastfeeding (promoting initiation: 1.36, 95% CI 1.14 to 1.61; *p* < 0.00001; exclusive breastfeeding: RR 2.78, 95% CI 1.74 to 4.44; *p* < 0.0001), child morbidity (RR 0.86, 95% CI 0.75 to 0.99; *p* = 0.0) and mortality (RR 0.75, 95% CI 0.55 to 1.03; *p* = 0.07), but an insignificant increase in care seeking practice, based on meta analyses (22); whereas the other review found improved infant birth outcomes, such as increased infant weight, particularly among multilingual programmes (45).

### Screenings

Of the five reviews (Table 4) (46–50), two analysed task shifting, whereby nurses performed colorectal cancer and skin cancer screenings (46, 47). Three reviews analysed the roles of CHWs, one on mammography screening uptake (48), one on screenings for several types of cancer (49) and one on TB screening for hard-to-reach populations (50).

**TABLE 4:** Screening and vaccinations (various countries, search performed in 2021).

Skill-mix typology	Description of Intervention	Profession in intervention (I); comparison (C)	Nr. studies	ME	Setting	Country	Population	Outcomes	Source
Screening									
TS	Colorectal cancer screening performed by nurses compared with physicians	I: NP, endoscopic nurses	6	-	Outpatient settings	CA (1), US (5)	45 years or older	• Nurse-led endoscopy comparable to physicians in quality and safety, no complications with nurse- led colonoscopies or physician-led colonoscopies (all studies)	(46)
		C: Physician endoscopists, gastroenterologist, surgeon, gastrenterologist						• Nurses performed colonoscopies according to quality standards (1 study)	
								• Nurses detected polyps at similar rates to endoscopists (3 studies)	
								• Nurses detected significantly higher adenomas compared to physicians (2 studies)	
								• Higher patient satisfaction with nurse-led colonoscopies (3 studies)	
								• Lower costs in nurse-led group compared with physicians (Nurse: $183, Physician: $283)	
TS	Evaluating skin cancer detection skills of APN compared with physicians	I: APN, NP, dermatology nurse	12	-	Primary care	Not specified	Males and females undergoing examination for skin cancer	• High sensitivity to identify malignant lesions by NPs (100%) (1 study), dermatology nurses showed less sensitivity (88%, 95%, CI 80–97) compared with general dermatologists (89%, 95% CI 83–96) (1 study) and dermatologists with expertise in skin cancer (100%, 95% CI 91–100) (1 study)	(47)
		C: Physician, dermatologist						• APNs’ ability to recognize suspicious or benign lesions was inconsistent, but showed improvement after training	
RE	CHW intervention on mammography. screening	I: CHW, health educators, peers, volunteers	24	yes	Various settings, outpatient clinics, other	US	Women 40 years of age or older without a history of breast cancer	• Pooled data showed significant effect in mammography screening rates (RR: 1.06, 95% CI: 1.02–1.11, p = 0.003)	(48)
		C: Usual care: profession not defined						• Sub-groups: in RCTs, significant improvements in screening rates (RR: 1.07; 95% CI: 1.03–1.12, p = 0.0005) but not from only quasi-experimental studies (RR: 1.03; 95% CI: 0.89–1.18, p = 0.71)	
								• In RCTs, recruitment from medical settings (RR: 1.41; 95% CI: 1.09–1.82, p = 0.008), programmes in urban settings (RR: 1.23; 95% CI: 1.09, 1.39, p = 0.001), and programmes tailored to the needs of ethnic minority groups (RR: 1.58, 95% CI: 1.29–1.93, p = 0.0001) showed more pronounced effects	
RE	CHW interventions on cancer screenings (and other areas)	I: CHWs	30	no	Community-based, home	US (29), IN (1)	At risk groups	• All cancer: Improvements in cancer screening (21 out of 30 RCTs)	(49)
		C: usual care, professions not defined						• Breast cancer: significant improvements (6%–33% increase) in mammography screening (9 of 16 RCTs)	
								• Cervical cancer: significantly increased participation in Pap smear tests (7%–29% increase) (9 of 16 RCTs)	
								• Only 3 studies on colorectal cancer screening, of which one showed statistically significant results (1 of 3 studies)	
RE	Skill-mix and service delivery models for TB prevention	I: Various professions, primarily CHWs, peers, lay workers	5	no	Various, mobile TB clinics, community	UK (2), DE (1), PT (1), ES (1)	Vulnerable and hard-to-reach groups	• Limited evidence, but suggests to involve CHWs from the same (migrant) community to improve TB screening uptake; moreover street teams and peers were also shown to improve TB screening by providing health education, promoting screening and organizing contract tracing	(50)
		C: usual care						• Outreach teams (e.g., mobile TB clinics) may improve TB screening uptake	
Vaccination									
O (RE + TS)	Pharmacists providing influenza vaccination on vaccination rates	I: Pharmacists	11	no	Various settings	US (5), UK (3), CA(3)	All adults (with sub-group analysis for adults >65)	• Allowing pharmacists to undertake influenza vaccination was associated between an 10% increase in vaccination rates (one study) and no discernible effect (3 studies)	(51)
		C: usual care (no vaccinations provided or substituting physicians)						• Pharmacists with the most autonomy demonstrated the largest rate increases	
O (RE + TS)	Lay health worker (LHW) contribution to vaccination uptake among children	I: LHWs (with some training)	4	Yes	Various PC settings, home visits, community	US (3), IE (1)	Children under age of 2 years with vaccination not being up to date	• LHWs promoting immunization uptake in children (RR 1.22, 95% CI 1.10 to 1.37; p = 0.0004) (I2 = 58%, p = 0.07); based on 4 studies with limited heterogeneity	(22)
		C: n/r							

Notes: APN, Advanced Practice Nurse; CHW, Community Health Worker; LHW, Lay Health Worker; NP, Nurse Practitioner, O=Hybrid/various/other; PC, Primary Care; RCT, Randomised Control Trail; RE, Role Expansion; TB, Tuberculosis; TS, Task Shifting. Country abbreviations: CA, Canada; DE, Germany; ES, Spain; IE, Ireland; IN, India; PT, Portugal; UK, United Kingdom; US, United States of America.

Of the reviews on task-shifting from physicians to nurses, there were no differences in the quality of care in colorectal cancer screenings (endoscopies, colonoscopies) provided by Advanced Practice Nurses or other specialized nurses (e.g., endoscopic nurse) compared with physicians (e.g., endoscopists, gastroenterologists) (46), but significantly higher levels of adenomas detected by these nurses compared with physicians. Patient satisfaction was higher in the nurse-led groups. For skin cancer screenings performed by specialized nurses, results were mixed by nurses’ qualification and training (47). Nurse Practitioners if adequately trained were shown to be able to identifiy malignant lesions with equal levels of sensitivity to dermatologists with expertise in skin cancer and higher levels of sensitivity than dermatologists. However, dermatology nurses showed lower sensitivity (ibid.).

A review on CHWs and screenings found statistically higher mammography screening rates when CHWs provided, e.g., outreach, education, home visits, sessions in communities (RR: 1.06, 95% CI: 1.02–1.11, *p* = 0.003), with sub-group analyses showing more pronounced effects for programmes targeting ethnic minorities, participants recruited from medical settings and in urban areas (48). In a review of various screenings, CHWs performed education, counseling, case management, navigation assistance, facilitated access and social support, which were delivered in collaboration with or supervised by other health professionals (49). The review found significant improvements in screening uptake for mammography (6%–33% increase) and cervical cancer (7%–29% increase in pap smear tests) compared to usual care. The review on tuberculosis prevention (50) focused on hard-to-reach populations, services were performed by various professions, but with a focus on CHWs, especially from the same migrant community, street teams, peers and outreach teams. It showed improved TB screening uptake with health education, promoting screening uptake and organizing contract tracing, yet based on limited evidence (ibid.).

### Vaccinations

The two reviews on vaccinations (Table 4) covered task-shifting and role expansion with limited delination of the two concepts (22, 51). The review on community pharmacists (51) found that influenza vaccination rates varied but pharmacists with greater autonomy showed higher vaccination rates. There was some evidence of a small effect of pharmacists substituting for physicians which also impacts on vaccination rates.

The meta-analysis on the role of lay health workers in promoting access to immunization (through home visits, postcards or phone calls, or both) for specifically for children (under age of 2 years) whose immunization schedules were not up to date or who had not received any vaccinations (22) found a statistically significant increase in immunization uptake in children (RR 1.22, 95% CI 1.10 to 1.37; *p* = 0.0004) compared to usual care.

### Skill-Mix and Lifestyle Modification

The 18 systematic reviews were grouped following SNAPW (Table 5). One review addressed skill-mix changes on smoking cessation (52), four reviews evaluated skill-mix and lifestyle change related to healthy nutrition (53–56), two covered physical activity (57, 58), five focused on obesity prevention (59–63) and six covered various interventions (64–69).

**TABLE 5:** Skill-mix with focus on SNAPW lifestyle modification (various countries, search performed in 2021).

Skill-mix typology	Description of Intervention	Profession in intervention (I), comparison (C)	Nr. studies	ME	Setting	Country	Population	Outcomes	Source
Smoking									
RE	Smoking cessation interventions in community pharmacy. Interventions involved providing advice and counseling	I: Community pharmacists	5	yes	Community pharmacy	US, UK, SE	Smoking population	• ME showed improved abstinence rates (RR 2.21, 95% CI: 1.49–3.29) (5 studies)	(52)
		C: usual care/no care						• Nicotine replacement therapy plus counseling showed better abstinence rates	
Nutrition									
RE	Minimum one face-to-face individualised consultation on nutrition care aimed at supporting individual to modify their dietary behaviors including any or all components of the Nutrition Care Process (i.e., nutrition assessment, nutrition diagnosis, nutrition intervention, and nutrition monitoring and evaluation)	I: Dieticians	26	no	Primary healthcare settings	US (8), AU (3), UK (3), Hong Kong (2), other	Adult patients, often with risk factors	• 18/26 studies with statistically significant differences in dietary, anthropometric, or clinical indicators between intervention and comparators	(53)
		C: n/r						• 4/4 studies with statistical improvements on glycemic control, 4/4 on dietary change, 4/7 on anthropometry, 2/8 on cholesterol, 1/5 on triglycerides, 0/3 on blood pressure	
								• Dietetic consultation effective in 11/21 studies for minim. one indicator (blood pressure, blood lipid and glucose levels), 7/20 studies on anthropometric data (weight, BMI, waist circumference), 8/12 studies on dietary data (energy, carbohydrate, protein, fat, sodium, calcium, vitamin C)	
RE	Dietary advice to reduce blood cholesterol given by a dietician or nutritionist versus other health professional or self-help resources	I: Dietician, nutritionist	12	yes	Primary care settings, workplace, outpatient clinic seetings	UK, USA, AU	Adults with or without existing heart disease or previous myocardial infarction	• ME: Higher reduction in blood cholesterol among dietitican-delivered advice group than by physician (change: −0.25 mmol/L (95% CI −0.373737, −0.12 mmol/L))	(56)
		C: Other (physician: 4, nurse: 1, counsellor: 1) or self-help (7 studies)						• No statistically significant difference in blood cholesterol between dietitians and self-help resources (−0.10 mmol/L (95% CI −0.22, 0.03 mmol/L))	
RE	Interventions on healthy diet in primary care, including dietary counselling, motivational interviews, advice for behaviour change	I: physicians/GPs, nurses, nutritionist, health educator, others n/r	10	yes	PC settings (general practice, university clinical centre, family practices)	US (6), UK (2), IT (1), JP (1)	Healthy adults	• ME: significant increase in fruit consumption of 0.25 (95% CI: 0.01 to 0.49, p = 0.04) and vegetable consumption of 0.25 (95% CI 0.06 to 0.44, p = 0.04) servings/day	(54)
		C. n/r						• Significant increase of dietary fibre: 1.97 (95% CI 0.43 to 3.52, p = 0.012) gm per day	
								• Significant mean decrease in fat intake of 5.2% of total energy (95% CI -1.5% to -8.8%, p = 0.005)	
								• Mean decrease in serum cholesterol of 0.10 (−0.19 to 0.00 mmol/L, p = 0.049)	
RE	Healthy nutrition (e.g., nutrition assessment, advice and nutrition counselling, referral to other nutrition-focused health professionals) to improve dietary behaviours	I: GPs (2), nurses (8), dieticians (3), health counsellors (1), working alone (15) or in team (6), C: n/r	21	no	PC	JP (1), US (3), UK (7), DK (1), AU (2), NZ (2), NL (3), FI (1), IT (1)	Adults	• 12 studies with significant improvements in participants’ dietary behaviours, e.g., increased daily consumption of fruit, vegetables, high-fibre bread and fish	(55)
								• 7 studies with no improvement in dietary behaviours; one observed equal improvements among participants in the intervention and control groups and one found a reduction in participants’ daily fruit and vegetable intake	
Physical Activity									
RE	Physiotherapist-led physical activity interventions (one-to-one, face-to-face) aimed at increasing physical activity levels among adults	I: Physiotherapist-led care	8	yes	Outpatient and primary care settings, clinic-based private practice	NL (4), NO (2), AU (1), DE (1)	Adults with risk factors of or NCDs, musculoskeletal injury	• ME: Significantly increased physical activity (minimum recommended level) (OR 2.15, 95% CI, 1.35–3.43, p= 0.001)	(57)
		C: usual care/not consistently reported						• Significant effect on total physical activity in the short term (SMD 0.15, 95% CI, 0.03–0.27, p = 0.02) but not in the long term	
RE	Physical activity promotion to increase activity or fitness levels (or both). Inverventions: advice or counselling face to face or by phone (or both) in two or more sessions, supported with written materials or reminders (e.g., by phone)	I: PC physician, nurse, physiotherapist; health visitor, trained facilitator	15	yes	PC settings, PC and sports facility, home	UK (6), NZ (3), US (2), CH (1), NL (1), AU (1), CA (1)	sedentary adults, recruited in PC	• Physical activity promotion sessions: small to medium positive effects (OR 1.42, 95% CI 1.17 to 1.73; SDM 0.25, 0.11 to 0.38) (13 trials, self reported physical activity)	(58)
		C: no/usual care						• Exercise referral: small non-significant effects on self reported physical activity (OR 1.38; 0.98 to 1.95; SDM 0.20, −0.21 to 0.61)	
								• Cardiorespiratory fitness: medium non-significant positive effect (SDM 0.51, −0.18 to 1.20) (3 trials)	
Weight									
Re	Nurse obesity prevention interventions in schools	I: Registered Nurses/School nurses	11	Yes	Schools	US (8), Europe (2), Asia (1)	School children (healthy and obese)	• Small but significant decreases in children’s weight, measured by BMI or BMIz	(59)
	• All children: 7 studies	C: no intervention/usual care (e.g., leaflets, other)						• Significant decreases in BMI (6 studies: SMD: −0.48, 95% CI: −0.84, −0.12), BMIz (5 studies: −0.10, 95% CI: −0.15, −0.05), and BMI percentile (3 studies: −0.41, 95% CI: −0.60, −0.21)	
	• Focus on overweight/obese children: 4 studies. Interventions included education & counseling, weight management, motivational interviewing, physical activity, nutrition, parent involvement								
RE	Nurse delivered lifestyle intervention to reduce NCD risk factors associated with obesity. Involving: behavioural counselling in an appointment (5–30 min), using behaviour change techniques, e.g., stage matching, motivational interviewing or goal setting	I: Nurses (NPs, practice nurses, public health nurses, community nurses, health visitors)	28	no	Primary healthcare setting (e.g., general practice, community health centre)	UK (9), US (13), FI (4), NL (1), NZ (1)	Adults	• Significant improvements: weight reduction (6 studies) or control (1), systolic BP (3), diastolic BP (1), cholesterol (6), improved dietary intake (12), fitness (1), PA (3), anthropometry (1)	(60)
		C: other health professionals						• Counselling by nurses more effective than health screening (10 studies)	
								• Counselling based on behaviour change theory more effective than non-behavioural counselling (*n* = 3)	
								• High dose of behavioural counselling improved patient satisfaction (8)	
RE	Nurse-delivered weight management interventions across the life span. Interventions involved consultations with goal setting, motivational interviewing or coaching, and/or lifestyle change education	I: Nurses alone or in teams	20	no	Outpatient clinical settings (58% of interventions), workplace, schools, child care facilities	US (6), NL (5), AU (3), NO, FI, RU, SE, UK, TR, TW (1 study each)	Overweight individuals including children and parents	• Significantly reduced BMI or weight reported in 65% of the studies	(61)
		C: various professions						• Particularly successful in reducing BMI or weight: nurses promoting health promotion activities, operating within multidisciplinary teams and/or providing consultations, physical activity education, and coaching over the phone	
RE	Analysis of practice nurses' role in lifestyle counselling regarding weight management in primary care and their cooperation with other health professionals	I: Practice nurses (e.g., NP, primary care nurse)	45	no	Primary Care	Majority in Europe (33, of which 18 in UK; 10 in NL), plus Australia (6), US (5)	Adults	• Weight: RCTs: 10/12 with positive outcomes, other study designs: less consistent results	(62)
		C: n/r (not consistent), some RCTs with GPs as comparators						• Practice nurses more often discussed diet and physical activity than GPs	
								• Nurses achieved equally good health outcomes compared to GPs	
								• Nurses took longer in their consultations than GPs, and increased patient satisfaction	
RE	Interventions to change the behaviour of health professionals or the organisation of care to promote weight reduction in children and adults with overweight or obesity	I: Doctors (GP and specialist), dieticians, nurses, practice nurses, consultants	12	no	Family practices	US (7), UK (3), AU (2)	Adults and children with overweight or obesity	• Adults may lose more weight if care provided by a dietician (by −5.60 kg, 95% CI −4.83 kg to −6.37 kg) or by a doctor-dietician team (by −6.70 kg, 95% CI −7.52 kg to −5.88 kg; 1 study, N = 270 adults; low-certainty evidence). Shared care with little or no difference in the BMI z-score of children with obesity (adjusted MD −0.05, 95% CI −0.14 to 0.03; 1 study, N = 105 children; low-certainty evidence)	(63)
		C: usual care						• Cost effectiveness: N = 2 studies, one study achieved weight loss at a modest cost in both intervention groups (doctor and doctor-dietician). One study favoured mail and standard care over telephone consultations	
Various/Mulitple lifestyle									
TS	Interventions covered secondary prevention and disease management of patients following clinical guidelines	I: Nurses, primarily NPs	12	No	Primary care settings	NL, RU, UK, ZA	Patients attending primary care settings, primarily with chronic conditions	• Majority of outcome measure (84%) with no significant differences between nurse-led and physician-led care	(68)
		C: Physicians						• Nurse-led care showed better outcomes in the secondary prevention of heart diseases, managing dyspepsia; and lowering CVD risk in diabetic patients	
								• Significantly reduced stroke risk and CHD risk	
RE	Interventions delivered in community pharmacies for alcohol reduction, smoking cessation and weight management	I: Community pharmacist, pharmacy technician or medicines counter assistant		Yes (smoking)	Community pharmacy	UK (8), US (4), AU (2), CA (1), DK (1), JP (1), NL (1), TH(1)	Adults	• Smoking cessation (12 studies): Behavioural support and nicotine replacement therapy are effective (pooled OR of 2.56 (95% CI 1.45 to 4.53) and cost-effective for active interventions vs. usual care	(69)
		C: Ususal care, other control group						• Weight management (5 studies): Pharmacy-based interventions produced similar weight loss at similar provider cost compared with active interventions in other primary care settings in the short term (up to 6 months follow-up) but not longer term	
								• Alcohol reduction (2 studies): Insufficient evidence on effectiveness and cost-effectiveness	
RE	Health behaviour change, delivered by physical therapists. Interventions: counseling, goal setting to increase physical activity (or other), strategy development, support, identifying barriers, introduction into self-management	I: Physical therapists alone or with multiprofessional team	7	No	PC setting	AU, FI, NL, SE, US	Adults physically inactive or with lifestyle-related risk factor or condition	• Significant improvements in (duration of weekly) physical activity or (home) exercise (3 studies), decrease in weight (2 studies), diastolic blood pressure (1 study), stress counselling (1 study each)	(67)
		C: n/r						• Self-monitoring of physical activity increased physical activity (2 studies)	
								• Improvements for cardiovascular risk factors (1 study)	
RE	PC provider roles in health literacy for individuals to make SNAPW decisions for at risk groups for developing chronic conditions. Interventions: motivational interviewing, counselling, written material, group education, computer-assisted interventions	I: Multi-disciplinary team (N = 11), physicians (N = 9), lay worker (N = 6), educator (N = 6), nurse (N = 5), electronic interventions (N = 7), others	52	No	PC settings, community, other	USA (30), UK (7), plus AU, NZ, SE, CH, NL, CA, JP	Adults with at least one SNAPW risk factor	• 71% of the studies (37/52) with statistically improved health literacy	(64)
		C: n/r						• Health literacy and SNAPW risk factor were both improved for 61% (14/23) of interventions to address nutrition, 54% (15/28) for PA, 43% (3/7) for weight and 40% (6/15) for smoking	
								• By professions: 92% (11/12) of studies provided by nurses, dieticians or educators showed improvements, 91% (10/11) if provided by multidisciplinary teams and 33% (3/9) of studies provided by physicians	
								• Physicians tended to provide low density interventions vs. medium/high density by nurses/others	
O	Interdisciplinary interventions such as health education delivered to individuals or groups, e.g., physical activity, stress management, counselling, individualised training, life style advice. Interventions delivered from multidisciplinary teams	I: Physicians, nurses, dieticians, physiotherapists, psychologists, pharmacists	16	No	Primary setting	Not reported	Adults with or at risk of diabetes, overweight/obesity, hypertensions/CVD risk factors	• Significant reduction among interdisciplinary interventions in anthropometric indices (7/10 RCTs)	(66)
		C: physicians, dieticians						• Significant decrease in blood pressure (6/7 studies)	
								• Mixed results in blood glucose improvements (2/7), blood lipids (1/6 studies)	
								• Significant weight reduction of community-based interventions over time (5/6 studies)	
RE	Health-related lifestyle advice provided by lay health workers, aimed at individuals or groups with the aim of health improvement. Interventions comprised: HIV prevention; healthy diet, physical activity; breastfeeding; mental health, chronic disease management; smoking cessation	I: Trained, but generally unqualified health-related lifestyle advisors (paid workers or volunteers)	24	No	n/a (probably community setting)	Not reported	Marginalised populations, cancer survivors, poor/urban patients with diabetes, immigrants	• Cost-effectiveness of health-related lifestyle advice (HRLA) in 24 trials. Little evidence of effectiveness of lifestyle advice for exercise/improved diets	(65)
		C: n/r						• Cost-effectiveness varied: incremental cost effectiveness ratios at £ (GBP) 6,000 for smoking cessation; £ 14,000 for a telephone based type 2 diabetes management; and £ 250,000 or greater for promotion of mammography attendence and for HIV prevention amongst drug users	

Notes: SNAPW: Smoking, Nutrition, Physical Activity, Weight management/reduction, BMI, Body mass index; BMIz, Body Mass Index (“z” for children), BP, Blood pressure; CHD, Coronary heart disease; CVD, Cardiovascular disease; GP, General practitioner; HIV, human immunodeficiency virus; ME, Meta Analysis; NCD, Noncommunicable Disease; PC, Primary Care, O=Hybrid/various/other; RCT, Randomized Control Trial; RE, Role Expansion; SNAPW, Smoking, Nutrition, Physical Activity, Weight reduction/management; TS, Task Shifting. Country abbreviations: AU, Australia; CA, Canada; IT, Italy; JP, Japan; DK, Denmark; AU, ; NL, The Netherlands; FI, Finland; SE, Sweden; NO, Norway; DE, Germany; RU, Russia; CH, Switzerland; NZ, New Zealand; UK, United Kingdom; IN, India; MX, Mexico; TH, Thailand; TR, Turkey; TW, Taiwan; ZA, South Africa; US, United States of America.

### Smoking

The meta-analysis on community pharmacists providing smoking cessation interventions (either one-to-one counseling or group sessions) to smokers coming to the pharmacy (52) found significantly improved abstinence rates (RR: 2.21, 95% CI: 1.49–3.29) compared to usual or no care.

### Nutrition/Diet

Of the four reviews, two analysed the roles of dieticians (53, 56), the others covered multiple professions (54, 55). The two reviews assessing the role of dieticians found that the majority of included studies showed statistically significant improvements favouring the intervention provided by dieticians. In one review, the majority of studies showed significant improvements in dietary, anthropometric or clinical indicators (blood pressure, serum measures, including cholesterol, triglycerides, and sodium; and blood glucose measures), the comparison groups entailed groups which received no intervention, usual care (medical care that did not include nutrition care from any health professional) or minimal care (attendance at a single general nutrition session or provision of a diet sheet) (53). A meta analysis (56) found that dietary advice and related services provided by dieticians significantly lowered blood cholesterol in the short to medium term compared with doctors (−0.25 mmol/L 95% CI: −0.373,737, −0.12 mmol/L). Results were not statistically different between dieticians and self-help resources and there was no evidence that dieticians were better than nurses or other professions, based on the small number of studies included.

The reviews covering interventions targeting diet by multiple professions (e.g., GPs, physicians, nurses, nutritionists) found generally positive outcomes on dietary behaviour (54, 55), of which a meta analysis (54) showed statistically significant increases in fruit (0.25 95% 95% CI: 0.01 to 0.49, *p* = 0.04) and vegetable consumptions (0.25 95% CI: 0.06 to 0.44, *p* = 0.01) and dietary fibre among healthy adults, and decreases in total fat intake (5.2% of total energy 95% CI: 1.5%–8.8%, *p* = 0.005), as well as positive changes in serum cholesterol compared to usual care or no intervention.

### Physical Activity

Of the two systematic reviews, one showed that physiotherapist-led one-on-one counselling significantly improved physical activity levels compared to usual care (OR 2.15, 95% CI, 1.35–3.43, *p* = 0.001) (57). Orrow, Kinmonth (58) found statistically significant small to medium positive effects on adults’ self-reported physical activity levels (OR 1.42, 95% CI 1.17 to 1.73; SDM 0.25, 0.11–0.38), when primary care professionals provided advice or counselling in two or more sessions, whereas referrals found non-significant effects.

### Weight Reduction and Obesity Prevention

Of the five reviews (59–63), four covered the roles of nurses (59–62) and one subsumed various professionals (63). Interventions ranged from nurses delivering obesity prevention activities in schools (59), in primary care settings (60–62) and various interventions to promote weight reduction in children and adults with overweight (63).

The reviews on nurse-delivered interventions found statistically improved weight reduction of school children (BMI, SMD: −0.48, 95% CI: −0.84, −0.12) delivered in school settings compared to usual care (e.g., leaflets) or no interventions (59); on adults’ weight, blood pressure, cholesterol, dietary intake (60) and on BMI in 65% of studies compared to interventions provided by other health professionals (61). Positive outcomes were associated with nurses providing individual counselling sessions compared to usual care or health screening (60–62), long-term follow-up assessments compared to GPs (62), health promotion activities, education on physical activity and working within multidisciplinary teams (61).

A review on obesity prevention (63) analysed a wide range of interventions expanding roles or organization of care. The largest effect on adult weight reduction had dietician-delivered interventions (Mean difference (MD): −5.60 kg, 95% CI −4.83 kg to −6.37 kg) or a doctor-dietician shared care team (MD: −6.70 kg, 95% CI −7.52 kg to −5.88 kg), whereas providing education to GP’s, clinical decision tools or increased GP compliance to guidelines led to little or no difference in weight. Two studies investigated costs of which one showed that weight lost was achieved at modest costs in doctor-dietician and doctor-led interventions compared with usual care.

### Various Lifestyle-Related Interventions

Of the six reviews (64–69), one analysed task-shifting from physicians to nurses (68), one evaluated extending roles among community pharamacists (69), two evaluated extended roles among various professionals or teams (64, 66), one review covered the roles of physical therapists (67) and one lay workers (65).

In the review on task-shifting from physicians to nurses (68), the interventions were delivered primarily by Nurse Practitioners, following clinical protocols. Nurse-led care was comparable to physician-led care for the majority of outcomes (84%). For the remainder 16%, nurse-led care showed statistically significant improvements, for instance preventing heart diseases or lowering CVD risk among patients with diabetes.

The review on role expansions of community pharamcists (69) covered lifestyle advice on smoking, weight and alcohol consumption; it showed that behavioural support and nicotine replacement therapy interventions were more effective (OR 2.56, 95% CI 1.45–4.53) and cost-effective compared to non-active control, usual care, or other intervention. Pharmacist-delivered weight loss interventions led to similar weight loss as interventions in other primary care settings in the short-term (up to 6 months) with similar provider costs. Longer term effects on weight loss were inconclusive. On interventions targeting reduction of alcohol intake, there was insufficient evidence on effectiveness and cost-effectiveness.

The review on expanded roles of physical therapists in lifestyle advice showed significant improvement in physical activity, weight, blood pressure and cardiovascular risk factors compared to usual care, but based on small numbers of studies (67). Studies with multiple intervention components and several behavioural sessions (instead of one-off exercise prescription or single counselling) were associated with improved outcomes. Yet, many studies analysed short-term effects instead of longer-term outcomes.

A review on primary care professionals providing health literacy interventions (64) reported improvements in 71% of the studies. Interventions varied and included motivational interviewing, counselling, written material, group education, computer-assisted interventions, and multiple interventions. A review on interdisciplinary interventions with behavioural elements on lifestyle changes and effects on SNAPW risk factor outcomes (66) showed mixed results, improvements were identified for weight loss, but not for blood lipids, glucose and blood pressure. Interventions were more effective if provided by collaborations between dieticians, exercise physiologists, psychologists and intensive patient engagement compared to usual care provided by physicians or dieticians only, but required ongoing patient support for long-term effect (66).

The cost-effectiveness analysis on lay workers providing health-related lifestyle-related advice to poor and marginalized groups and cancer survivors, among others, covered various interventions including smoking cessation, diabetes management and HIV prevention among drug users. It found large differences in the cost effectiveness ratios of the interventions (incremental cost ratios estimated at 6,000 GBP for smoking cessation, up to 250,000 GBP or higher for mammography and for HIV prevention) (65).

## Discussion

A total of 31 systematic reviews were identified on skill-mix changes focused on health promotion and prevention interventions and outcomes. Seven covered MCH, five screenings and two vaccinations. The majority (N = 18) analysed skill-mix targeting lifestyle modification. There was large variability in the number of reviews available by prevention area and their quality, as demonstrated by the AMSTAR II scores.

New skill-mix roles in MCH were home visitors who often focused interventions on vulnerable or at-risk groups. The results indicated several positive outcomes for mothers or parents, particularly if aimed at women at risk, if provided by health professionals (nurses, social workers) in teams and *via* bundled interventions. Previous research on patient navigators performing home visits for people with cancer, other chronic conditions, transitional care or on screenings, found improved access, reduced hospital readmissions and improved patient outcomes (37).

Regarding screenings, the included reviews suggested that Advanced Practice Nurses can provide safe and effective screening of a similar quality to physicians for most measures in colorectal cancer and skin cancer, if adequately trained. A Cochrane review on nurses substituting for physicians found similar results in primary care and chronic care, however, it covered all nurses (70), and information on educational background was not always sufficiently available. In our overview of reviews, CHWs were shown to contribute in supportive roles by educating, awareness raising and other interventions and showed improved uptake of cancer screening, particularly among ethnic minorities and hard to reach groups. However, AMSTAR II grading was low for many reviews on screening.

On vaccination, we identified only two reviews. The one on pharmacists suggests that enabling pharmacists to administer vaccines can improve vaccination rates when given greater autonomy. The other review found that lay workers can promote vaccine uptake. No systematic reviews were identified on other professions. In addition, the majority of evidence on screening and vaccinations were from the US, Canada and UK. The experiences of countries during the COVID-19 pandemic (16, 17) allowing non-medical professions to vaccinate, often under pandemic laws, should be evaluated as to the effectiveness, efficiency and scalability in different contexts.

Many of the 18 reviews on lifestyle modification were of high quality, demonstrated by the highest AMSTAR II sum score compared with the other prevention areas. Community pharmacists were suggested to be effective in promoting smoking cessation. Nurse- or dietician-delivered interventions were shown promising to support weight-management. Nurse-delivered interventions were effective when delivered autonomously or within teams, in schools or primary healthcare settings. Interventions led by physiotherapists or other primary care professionals were suggested effective at promoting physical activity. It should be noted, however, that many studies did not analyse long-term effects.

In terms of skill-mix typology, we identified 15 reviews on role expansions, four on task-shifting and 12 “other” (mix of typologies, various, not clearly identifiable). The reasons for the difference in the number of reviews on role expansions vs. task-shifting is unknown. A Cochrane review on task-shifting from physicians to nurses found that little research existed for preventive services and health education (70). The difference may also suggest that skill-mix in health promotion and prevention remains a largely new area, hence reflects role expansion rather than task shifting. This is supported by previous research on providers (71), primary care practices (8, 72) and international skill-mix developments (25). Yet, the 12 reviews in our study identified as “other” suggest that the boundaries of skill-mix changes are not always clear-cut, in line with previous literature (11, 70, 73).

On the task-shifting typology, previous research showed the safety and effectiveness of task-shifting from physicians to nurses or pharmacists, e.g., for patients with chronic conditions (74, 75), and from physicians to nurses for all conditions in primary care (70) or for non-medical prescribing (76–79), if adequately trained.

The majority of reviews in our analysis covered multiple professions. Among the reviews covering single professions, most were on nurses, followed by pharmacists and lay workers. Similar findings were reported for skill-mix and chronic conditions, where the largest number of reviews covered nurses and pharmacists (74). In many countries, nurses’ and pharmacists’ scopes-of-practice has expanded over the last decade (15–17, 80, 81), including prescribing authority for nurses (82), and other clinical tasks (81), which has been reinforced by the COVID-19 pandemic in some countries (17, 83).

The number of systematic reviews in our analysis focusing on costs or cost effectiveness of skill-mix changes was small and showed no coherent results (40, 44, 63, 65, 69). The review on weight reduction showed that weight loss was achieved at modest costs in both intervention groups (doctor-dietician and doctor-led), compared with usual care but based on only one study (63). Two reviews found large variations in the interventions, their intensity, incremental costs and cost effectiveness (44, 65). The professions delivering the interventions varied (44), one review (65) covered lay workers and peers. All reviews were based on small numbers of studies or reported several limitations (40, 44, 63, 65, 69), which is in line with previous research in primary care, for instance for nurses substituting for physicians (70) and various new professional roles (84).

Several of the included reviews covered skill-mix changes taking place in people’s homes, communities or other settings (e.g., schools), suggesting that the primary care settings are diversifying. Skill-mix changes have been identified as one of the levers for re-orienting primary care services from curative care to integrated services (5). Implementation requires changes to the skills and roles of the professions involved, team and organizational changes, as well as changes to policy and financing (85). Education and training has been identified as critical (86, 87). A systematic review found that professional training may lead to a small but significant change in health professionals’ skills and behaviour for up to 12 months, which may subsequently change patients’ health behaviour (88). More research is needed on training contents, uptake in clinical practice and other interventions to achieve long-term effects among individuals and population groups.

### Limitations

This overview of reviews faces several limitations. First, we did not search for individual primary studies, nor did we examine overlap across the included systematic reviews. Moreover, the number of included reviews and their quality was highly variable. Second, whereas several reviews covered nurses, followed by pharmacists and CHWs, few covered other professions or multiprofessional teams. Many reviews covered multiple professions, which limit the results to roles or functions, but not by profession. Third, the exact roles and tasks performed were not always sufficiently reported. Moreover, while we used a skill-mix typology according to pre-defined criteria, it was not always possible to delineate between role expansion and task-shifting, which may have led to an overreporting of the category on “other”. Fourth, there were few reviews on costs. Future research should analyse at more detailed levels which professions with which education, skills and roles are most effective and cost-effective for specific prevention roles.

### Conclusion

Several promising skill-mix innovations were identified. They include expanded roles on lifestyle change, outreach roles and task-shifting. Yet, the quality of the evidence varied. There was inconclusive evidence on costs. More research is required on the educational requirements for new skill-sets by professions and upatek in practice to enhance links between primary care and population health.
