# Supplementary material for: Skill-Mix Changes Targeting Health Promotion and Prevention Interventions and Effects on Outcomes in all Settings (Except Hospitals): Overview of Reviews
Source: Int J Public Health. 2023 May 9;68:1605448. doi: 10.3389/ijph.2023.1605448 (PMC10203245; doi:10.3389/ijph.2023.1605448)
Supplement: Supplementary file 1 [file DataSheet1.docx]

**SUPPLEMENT**

**Supplement 1: Search strategy**

**EMBASE**

('skill mix'/de OR 'role change'/de OR 'task shifting'/de OR 'teamwork'/de OR 'case management'/de OR 'case manager'/de OR 'shared care'/de OR 'professional delegation'/de OR 'skill mix change'/de OR 'care coordination'/de OR 'pharmacist intervention'/de OR 'multi disciplinary team'/de OR 'shared decision making'/de OR ((skill* NEAR/3 mix) OR ((chang* OR multidisciplin* OR multi-disciplin* OR interdisciplin* OR inter-disciplin*) NEAR/3 (role* OR collaborat* OR cooperat*)) OR ((collaborat* OR cooperat*) NEAR/6 (doctor* OR physician* OR nurse* OR pharmacist* OR specialist* OR care OR healthcare)) OR 'new role*' OR ((task* OR decision*) NEAR/3 (shift* OR reallocat* OR allocat* OR sharing OR substit*)) OR teamwork OR (team NEXT/1 (work OR approach OR member* OR training OR educat* OR interact*)) OR (( multidisciplin* OR multi-disciplin* OR interdisciplin* OR inter-disciplin*) NEAR/3 (team* OR round*)) OR ((Shift* OR liaison* OR coordinat*) NEAR/3 (care OR rore*)) OR ((change* OR extend* OR expand* OR transform*) NEAR/3 (responsib* OR skill* OR boundar* OR competenc* OR boundar*)) OR ((non-medical* OR nonmedical* OR nurse* OR pharmacist* OR 'nurse practitioner*' OR 'nurse specialist*' OR 'specialist nurse*' OR 'physician assistant*' OR 'medical assistant*' OR PA) NEAR/3 (prescri*)) OR ((case OR discharge* OR nurse* OR care) NEXT/1 (manag* )) OR ((service* OR skill* OR role* OR task* OR responsib*) NEAR/3 transfer*) OR ((nurse* OR pharmacist* OR physician-assistant* OR pa OR medical-assistant* OR dentist* OR dentalassistant* OR physiotherapist* OR physical-therapist*) NEXT/1 (led OR intervention* OR managed OR run OR directed)) OR (Substitut* NEAR/3 (doctor* OR physician* OR nurse* OR pharmacist* OR specialist*)) OR ((care OR healthcare) NEXT/1 coordinat*) OR delegation OR (exten* NEAR/3 role*) OR (professional* NEAR/3 (autonom* OR boundar*)) OR (role* NEAR/6 ('nurse practitioner*' OR 'nurse specialist*' OR 'specialist nurse*' OR 'physician assistant*' OR 'medical assistant*' OR 'palliative care*' OR 'end of life*' OR 'informal care*' OR 'family care*')) OR 'new role*' OR 'chang* role*' OR 'shared care' OR 'joined consult*' OR 'Patient navigat*' OR ((additional OR advanced OR new OR extended OR changed OR expanded OR supplementary OR joint OR shared OR sharing) NEAR/6 (task* OR role* OR skill* OR competenc* OR responsib*)) OR (Replace* NEAR/3 (care OR healthcare)) OR ((new OR expanded OR enlarged OR advanced) NEAR/3 (scope*-of-practice)) OR (shar* NEAR/3 decision*)):ab,ti) AND ('health care personnel'/exp OR 'health personnel attitude'/exp OR caregiver/exp OR (('health care' OR healthcare) NEAR/3 (personnel* OR staff* OR worker* OR workforce*) OR nurse* OR doctor* OR ((clinical OR health* OR care OR medical OR end-of-life) NEAR/3 (manpower* OR workforce* OR human-resource* OR personnel* OR professional* OR staff* OR worker* OR visitor* OR provider* OR assistant*)) OR Physician* OR general-practitioner OR doctor* OR consultant* OR nurse OR specialist* OR clinician* OR ((Advanced OR mid-level ) NEAR/3 (Pract* OR provider*)) OR physician-assistant* OR medical-assistant OR physiotherapist* OR physi*-therapist* OR occupational-therapist* OR midwif* OR midwiv* OR dentist* OR dental-staff OR pharmacist* OR pharmac*-technic* OR medical-assist* OR MD-extender* OR physician-extender* OR psychiatrist* OR psychologist* OR psychotherapist* OR Dietician* OR Dietitian* OR nutritionist* OR ((speech OR language) NEAR/3 (therapist* OR pathologist*)) OR logopaedist* OR logopedist* OR audiologist* OR ophthalmologist* OR optometrist* OR caregiver* OR carer* OR caretaker* OR (communit* NEAR/3 professional*) OR paramedic* OR gp OR gps OR ((practice* OR gp) NEAR/3 receptionist*)):ab,ti) AND ('systematic review'/de OR 'meta analysis'/de OR (((systematic*) NEAR/3 review*) OR meta-analys*):ab,ti) NOT ('case report'/de OR 'case report':ti) NOT ([Conference Abstract]/lim OR [Letter]/lim OR [Note]/lim OR [Editorial]/lim) AND [english]/lim

**Medline Ovid**

(Patient Care Team/ OR case management/ OR Delegation, Professional/ OR ((skill* ADJ3 mix) OR ((chang* OR multidisciplin* OR multi-disciplin* OR interdisciplin* OR inter-disciplin*) ADJ3 (role* OR collaborat* OR cooperat*)) OR ((collaborat* OR cooperat*) ADJ6 (doctor* OR physician* OR nurse* OR pharmacist* OR specialist* OR care OR healthcare)) OR new role* OR ((task* OR decision*) ADJ3 (shift* OR reallocat* OR allocat* OR sharing OR substit*)) OR teamwork OR (team ADJ (work OR approach OR member* OR training OR educat* OR interact*)) OR (( multidisciplin* OR multi-disciplin* OR interdisciplin* OR inter-disciplin*) ADJ3 (team* OR round*)) OR ((Shift* OR liaison* OR coordinat*) ADJ3 (care OR rore*)) OR ((change* OR extend* OR expand* OR transform*) ADJ3 (responsib* OR skill* OR boundar* OR competenc* OR boundar*)) OR ((non-medical* OR nonmedical* OR nurse* OR pharmacist* OR nurse practitioner* OR nurse specialist* OR specialist nurse* OR physician assistant* OR medical assistant* OR PA) ADJ3 (prescri*)) OR ((case OR discharge* OR nurse* OR care) ADJ (manag* )) OR ((service* OR skill* OR role* OR task* OR responsib*) ADJ3 transfer*) OR ((nurse* OR pharmacist* OR physician-assistant* OR pa OR medical-assistant* OR dentist* OR dentalassistant* OR physiotherapist* OR physical-therapist*) ADJ (led OR intervention* OR managed OR run OR directed)) OR (Substitut* ADJ3 (doctor* OR physician* OR nurse* OR pharmacist* OR specialist*)) OR ((care OR healthcare) ADJ coordinat*) OR delegation OR (exten* ADJ3 role*) OR (professional* ADJ3 (autonom* OR boundar*)) OR (role* ADJ6 (nurse practitioner* OR nurse specialist* OR specialist nurse* OR physician assistant* OR medical assistant* OR palliative care* OR end of life* OR informal care* OR family care*)) OR new role* OR chang* role* OR shared care OR joined consult* OR Patient navigat* OR ((additional OR advanced OR new OR extended OR changed OR expanded OR supplementary OR joint OR shared OR sharing) ADJ6 (task* OR role* OR skill* OR competenc* OR responsib*)) OR (Replace* ADJ3 (care OR healthcare)) OR ((new OR expanded OR enlarged OR advanced) ADJ3 (scope*-of-practice)) OR (shar* ADJ3 decision*)).ab,ti.) AND (exp Health Personnel/ OR exp Attitude of Health Personnel/ OR caregivers/ OR exp interprofessional relation/ OR ((health care OR healthcare) ADJ3 (personnel* OR staff* OR worker* OR workforce*) OR nurse* OR doctor* OR ((clinical OR health* OR care OR medical OR end-of-life) ADJ3 (manpower* OR workforce* OR human-resource* OR personnel* OR professional* OR staff* OR worker* OR visitor* OR provider* OR assistant*)) OR Physician* OR general-practitioner OR doctor* OR consultant* OR nurse OR specialist* OR clinician* OR ((Advanced OR mid-level ) ADJ3 (Pract* OR provider*)) OR physician-assistant* OR medical-assistant OR physiotherapist* OR physi*-therapist* OR occupational-therapist* OR midwif* OR midwiv* OR dentist* OR dental-staff OR pharmacist* OR pharmac*-technic* OR medical-assist* OR MD-extender* OR physician-extender* OR psychiatrist* OR psychologist* OR psychotherapist* OR Dietician* OR Dietitian* OR nutritionist* OR ((speech OR language) ADJ3 (therapist* OR pathologist*)) OR logopaedist* OR logopedist* OR audiologist* OR ophthalmologist* OR optometrist* OR caregiver* OR carer* OR caretaker* OR (communit* ADJ3 professional*) OR paramedic* OR gp OR gps OR ((practice* OR gp) ADJ3 receptionist*)).ab,ti.) AND (Meta-Analysis/ OR (((systematic*) ADJ3 review*) OR meta-analys*).ab,ti.) NOT (case report/ OR case report.ti.) NOT (letter OR news OR comment OR editorial OR congresses OR abstracts).pt. AND english.la.

**CINAHL EBSCOhost**

(MH Multidisciplinary Care Team OR MH case management OR TI ((skill* N2 mix) OR ((chang* OR multidisciplin* OR multi-disciplin* OR interdisciplin* OR inter-disciplin*) N2 (role* OR collaborat* OR cooperat*)) OR ((collaborat* OR cooperat*) N5 (doctor* OR physician* OR nurse* OR pharmacist* OR specialist* OR care OR healthcare)) OR new role* OR ((task* OR decision*) N2 (shift* OR reallocat* OR allocat* OR sharing OR substit*)) OR teamwork OR (team N1 (work OR approach OR member* OR training OR educat* OR interact*)) OR (( multidisciplin* OR multi-disciplin* OR interdisciplin* OR inter-disciplin*) N2 (team* OR round*)) OR ((Shift* OR liaison* OR coordinat*) N2 (care OR rore*)) OR ((change* OR extend* OR expand* OR transform*) N2 (responsib* OR skill* OR boundar* OR competenc* OR boundar*)) OR ((non-medical* OR nonmedical* OR nurse* OR pharmacist* OR nurse practitioner* OR nurse specialist* OR specialist nurse* OR physician assistant* OR medical assistant* OR PA) N2 (prescri*)) OR ((case OR discharge* OR nurse* OR care) N1 (manag* )) OR ((service* OR skill* OR role* OR task* OR responsib*) N2 transfer*) OR ((nurse* OR pharmacist* OR physician-assistant* OR pa OR medical-assistant* OR dentist* OR dentalassistant* OR physiotherapist* OR physical-therapist*) N1 (led OR intervention* OR managed OR run OR directed)) OR (Substitut* N2 (doctor* OR physician* OR nurse* OR pharmacist* OR specialist*)) OR ((care OR healthcare) N1 coordinat*) OR delegation OR (exten* N2 role*) OR (professional* N2 (autonom* OR boundar*)) OR (role* N5 (nurse practitioner* OR nurse specialist* OR specialist nurse* OR physician assistant* OR medical assistant* OR palliative care* OR end of life* OR informal care* OR family care*)) OR new role* OR chang* role* OR shared care OR joined consult* OR Patient navigat* OR ((additional OR advanced OR new OR extended OR changed OR expanded OR supplementary OR joint OR shared OR sharing) N5 (task* OR role* OR skill* OR competenc* OR responsib*)) OR (Replace* N2 (care OR healthcare)) OR ((new OR expanded OR enlarged OR advanced) N2 (scope*-of-practice)) OR (shar* N2 decision*)) OR AB ((skill* N2 mix) OR ((chang* OR multidisciplin* OR multi-disciplin* OR interdisciplin* OR inter-disciplin*) N2 (role* OR collaborat* OR cooperat*)) OR ((collaborat* OR cooperat*) N5 (doctor* OR physician* OR nurse* OR pharmacist* OR specialist* OR care OR healthcare)) OR new role* OR ((task* OR decision*) N2 (shift* OR reallocat* OR allocat* OR sharing OR substit*)) OR teamwork OR (team N1 (work OR approach OR member* OR training OR educat* OR interact*)) OR (( multidisciplin* OR multi-disciplin* OR interdisciplin* OR inter-disciplin*) N2 (team* OR round*)) OR ((Shift* OR liaison* OR coordinat*) N2 (care OR rore*)) OR ((change* OR extend* OR expand* OR transform*) N2 (responsib* OR skill* OR boundar* OR competenc* OR boundar*)) OR ((non-medical* OR nonmedical* OR nurse* OR pharmacist* OR nurse practitioner* OR nurse specialist* OR specialist nurse* OR physician assistant* OR medical assistant* OR PA) N2 (prescri*)) OR ((case OR discharge* OR nurse* OR care) N1 (manag* )) OR ((service* OR skill* OR role* OR task* OR responsib*) N2 transfer*) OR ((nurse* OR pharmacist* OR physician-assistant* OR pa OR medical-assistant* OR dentist* OR dentalassistant* OR physiotherapist* OR physical-therapist*) N1 (led OR intervention* OR managed OR run OR directed)) OR (Substitut* N2 (doctor* OR physician* OR nurse* OR pharmacist* OR specialist*)) OR ((care OR healthcare) N1 coordinat*) OR delegation OR (exten* N2 role*) OR (professional* N2 (autonom* OR boundar*)) OR (role* N5 (nurse practitioner* OR nurse specialist* OR specialist nurse* OR physician assistant* OR medical assistant* OR palliative care* OR end of life* OR informal care* OR family care*)) OR new role* OR chang* role* OR shared care OR joined consult* OR Patient navigat* OR ((additional OR advanced OR new OR extended OR changed OR expanded OR supplementary OR joint OR shared OR sharing) N5 (task* OR role* OR skill* OR competenc* OR responsib*)) OR (Replace* N2 (care OR healthcare)) OR ((new OR expanded OR enlarged OR advanced) N2 (scope*-of-practice)) OR (shar* N2 decision*))) AND (MH Health Personnel+ OR MH Attitude of Health Personnel+ OR MH caregivers OR MH Interprofessional Relations+ OR TI ((health care OR healthcare) N2 (personnel* OR staff* OR worker* OR workforce*) OR nurse* OR doctor* OR ((clinical OR health* OR care OR medical OR end-of-life) N2 (manpower* OR workforce* OR human-resource* OR personnel* OR professional* OR staff* OR worker* OR visitor* OR provider* OR assistant*)) OR Physician* OR general-practitioner OR doctor* OR consultant* OR nurse OR specialist* OR clinician* OR ((Advanced OR mid-level ) N2 (Pract* OR provider*)) OR physician-assistant* OR medical-assistant OR physiotherapist* OR physi*-therapist* OR occupational-therapist* OR midwif* OR midwiv* OR dentist* OR dental-staff OR pharmacist* OR pharmac*-technic* OR medical-assist* OR MD-extender* OR physician-extender* OR psychiatrist* OR psychologist* OR psychotherapist* OR Dietician* OR Dietitian* OR nutritionist* OR ((speech OR language) N2 (therapist* OR pathologist*)) OR logopaedist* OR logopedist* OR audiologist* OR ophthalmologist* OR optometrist* OR caregiver* OR carer* OR caretaker* OR (communit* N2 professional*) OR paramedic* OR gp OR gps OR ((practice* OR gp) N2 receptionist*)) OR AB ((health care OR healthcare) N2 (personnel* OR staff* OR worker* OR workforce*) OR nurse* OR doctor* OR ((clinical OR health* OR care OR medical OR end-of-life) N2 (manpower* OR workforce* OR human-resource* OR personnel* OR professional* OR staff* OR worker* OR visitor* OR provider* OR assistant*)) OR Physician* OR general-practitioner OR doctor* OR consultant* OR nurse OR specialist* OR clinician* OR ((Advanced OR mid-level ) N2 (Pract* OR provider*)) OR physician-assistant* OR medical-assistant OR physiotherapist* OR physi*-therapist* OR occupational-therapist* OR midwif* OR midwiv* OR dentist* OR dental-staff OR pharmacist* OR pharmac*-technic* OR medical-assist* OR MD-extender* OR physician-extender* OR psychiatrist* OR psychologist* OR psychotherapist* OR Dietician* OR Dietitian* OR nutritionist* OR ((speech OR language) N2 (therapist* OR pathologist*)) OR logopaedist* OR logopedist* OR audiologist* OR ophthalmologist* OR optometrist* OR caregiver* OR carer* OR caretaker* OR (communit* N2 professional*) OR paramedic* OR gp OR gps OR ((practice* OR gp) N2 receptionist*))) AND (TI (Meta-Analysis+ OR (((systematic*) N2 review*) OR meta-analys*)) OR AB (Meta-Analysis+ OR (((systematic*) N2 review*) OR meta-analys*))) NOT (MH case report OR TI "case report") NOT PT (letter OR news OR comment OR editorial OR congresses OR abstracts) AND LA english

**PsycINFO Ovid**

(case management/ OR ((skill* ADJ3 mix) OR ((chang* OR multidisciplin* OR multi-disciplin* OR interdisciplin* OR inter-disciplin*) ADJ3 (role* OR collaborat* OR cooperat*)) OR ((collaborat* OR cooperat*) ADJ6 (doctor* OR physician* OR nurse* OR pharmacist* OR specialist* OR care OR healthcare)) OR new role* OR ((task* OR decision*) ADJ3 (shift* OR reallocat* OR allocat* OR sharing OR substit*)) OR teamwork OR (team ADJ (work OR approach OR member* OR training OR educat* OR interact*)) OR (( multidisciplin* OR multi-disciplin* OR interdisciplin* OR inter-disciplin*) ADJ3 (team* OR round*)) OR ((Shift* OR liaison* OR coordinat*) ADJ3 (care OR rore*)) OR ((change* OR extend* OR expand* OR transform*) ADJ3 (responsib* OR skill* OR boundar* OR competenc* OR boundar*)) OR ((non-medical* OR nonmedical* OR nurse* OR pharmacist* OR nurse practitioner* OR nurse specialist* OR specialist nurse* OR physician assistant* OR medical assistant* OR PA) ADJ3 (prescri*)) OR ((case OR discharge* OR nurse* OR care) ADJ (manag* )) OR ((service* OR skill* OR role* OR task* OR responsib*) ADJ3 transfer*) OR ((nurse* OR pharmacist* OR physician-assistant* OR pa OR medical-assistant* OR dentist* OR dentalassistant* OR physiotherapist* OR physical-therapist*) ADJ (led OR intervention* OR managed OR run OR directed)) OR (Substitut* ADJ3 (doctor* OR physician* OR nurse* OR pharmacist* OR specialist*)) OR ((care OR healthcare) ADJ coordinat*) OR delegation OR (exten* ADJ3 role*) OR (professional* ADJ3 (autonom* OR boundar*)) OR (role* ADJ6 (nurse practitioner* OR nurse specialist* OR specialist nurse* OR physician assistant* OR medical assistant* OR palliative care* OR end of life* OR informal care* OR family care*)) OR new role* OR chang* role* OR shared care OR joined consult* OR Patient navigat* OR ((additional OR advanced OR new OR extended OR changed OR expanded OR supplementary OR joint OR shared OR sharing) ADJ6 (task* OR role* OR skill* OR competenc* OR responsib*)) OR (Replace* ADJ3 (care OR healthcare)) OR ((new OR expanded OR enlarged OR advanced) ADJ3 (scope*-of-practice)) OR (shar* ADJ3 decision*)).ab,ti.) AND (exp Health Personnel/ OR exp Health Personnel Attitudes/ OR ((health care OR healthcare) ADJ3 (personnel* OR staff* OR worker* OR workforce*) OR nurse* OR doctor* OR ((clinical OR health* OR care OR medical OR end-of-life) ADJ3 (manpower* OR workforce* OR human-resource* OR personnel* OR professional* OR staff* OR worker* OR visitor* OR provider* OR assistant*)) OR Physician* OR general-practitioner OR doctor* OR consultant* OR nurse OR specialist* OR clinician* OR ((Advanced OR mid-level ) ADJ3 (Pract* OR provider*)) OR physician-assistant* OR medical-assistant OR physiotherapist* OR physi*-therapist* OR occupational-therapist* OR midwif* OR midwiv* OR dentist* OR dental-staff OR pharmacist* OR pharmac*-technic* OR medical-assist* OR MD-extender* OR physician-extender* OR psychiatrist* OR psychologist* OR psychotherapist* OR Dietician* OR Dietitian* OR nutritionist* OR ((speech OR language) ADJ3 (therapist* OR pathologist*)) OR logopaedist* OR logopedist* OR audiologist* OR ophthalmologist* OR optometrist* OR caregiver* OR carer* OR caretaker* OR (communit* ADJ3 professional*) OR paramedic* OR gp OR gps OR ((practice* OR gp) ADJ3 receptionist*)).ab,ti.) AND (Meta Analysis/ OR (((systematic*) ADJ3 review*) OR meta-analys*).ab,ti.) NOT (case report/ OR case report.ti.) NOT (letter OR news OR comment OR editorial OR congresses OR abstracts).pt. AND english.la.

**Cochrane CENTRAL**

(((skill* NEAR/3 mix) OR ((chang* OR multidisciplin* OR multi-disciplin* OR interdisciplin* OR inter-disciplin*) NEAR/3 (role* OR collaborat* OR cooperat*)) OR ((collaborat* OR cooperat*) NEAR/6 (doctor* OR physician* OR nurse* OR pharmacist* OR specialist* OR care OR healthcare)) OR 'new role*' OR ((task* OR decision*) NEAR/3 (shift* OR reallocat* OR allocat* OR sharing OR substit*)) OR teamwork OR (team NEXT/1 (work OR approach OR member* OR training OR educat* OR interact*)) OR (( multidisciplin* OR multi-disciplin* OR interdisciplin* OR inter-disciplin*) NEAR/3 (team* OR round*)) OR ((Shift* OR liaison* OR coordinat*) NEAR/3 (care OR rore*)) OR ((change* OR extend* OR expand* OR transform*) NEAR/3 (responsib* OR skill* OR boundar* OR competenc* OR boundar*)) OR ((non-medical* OR nonmedical* OR nurse* OR pharmacist* OR 'nurse practitioner*' OR 'nurse specialist*' OR 'specialist nurse*' OR 'physician assistant*' OR 'medical assistant*' OR PA) NEAR/3 (prescri*)) OR ((case OR discharge* OR nurse* OR care) NEXT/1 (manag* )) OR ((service* OR skill* OR role* OR task* OR responsib*) NEAR/3 transfer*) OR ((nurse* OR pharmacist* OR physician-assistant* OR pa OR medical-assistant* OR dentist* OR dentalassistant* OR physiotherapist* OR physical-therapist*) NEXT/1 (led OR intervention* OR managed OR run OR directed)) OR (Substitut* NEAR/3 (doctor* OR physician* OR nurse* OR pharmacist* OR specialist*)) OR ((care OR healthcare) NEXT/1 coordinat*) OR delegation OR (exten* NEAR/3 role*) OR (professional* NEAR/3 (autonom* OR boundar*)) OR (role* NEAR/6 ('nurse practitioner*' OR 'nurse specialist*' OR 'specialist nurse*' OR 'physician assistant*' OR 'medical assistant*' OR 'palliative care*' OR 'end of life*' OR 'informal care*' OR 'family care*')) OR 'new role*' OR 'chang* role*' OR 'shared care' OR 'joined consult*' OR 'Patient navigat*' OR ((additional OR advanced OR new OR extended OR changed OR expanded OR supplementary OR joint OR shared OR sharing) NEAR/6 (task* OR role* OR skill* OR competenc* OR responsib*)) OR (Replace* NEAR/3 (care OR healthcare)) OR ((new OR expanded OR enlarged OR advanced) NEAR/3 (scope*-of-practice)) OR (shar* NEAR/3 decision*)):ab,ti) AND ((('health care' OR healthcare) NEAR/3 (personnel* OR staff* OR worker* OR workforce*) OR nurse* OR doctor* OR ((clinical OR health* OR care OR medical OR end-of-life) NEAR/3 (manpower* OR workforce* OR human-resource* OR personnel* OR professional* OR staff* OR worker* OR visitor* OR provider* OR assistant*)) OR Physician* OR general-practitioner OR doctor* OR consultant* OR nurse OR specialist* OR clinician* OR ((Advanced OR mid-level ) NEAR/3 (Pract* OR provider*)) OR physician-assistant* OR medical-assistant OR physiotherapist* OR physi*-therapist* OR occupational-therapist* OR midwif* OR midwiv* OR dentist* OR dental-staff OR pharmacist* OR pharmac*-technic* OR medical-assist* OR MD-extender* OR physician-extender* OR psychiatrist* OR psychologist* OR psychotherapist* OR Dietician* OR Dietitian* OR nutritionist* OR ((speech OR language) NEAR/3 (therapist* OR pathologist*)) OR logopaedist* OR logopedist* OR audiologist* OR ophthalmologist* OR optometrist* OR caregiver* OR carer* OR caretaker* OR (communit* NEAR/3 professional*) OR paramedic* OR gp OR gps OR ((practice* OR gp) NEAR/3 receptionist*)):ab,ti)

**Web of science**

TS=((((skill* NEAR/2 mix) OR ((chang* OR multidisciplin* OR multi-disciplin* OR interdisciplin* OR inter-disciplin*) NEAR/2 (role* OR collaborat* OR cooperat*)) OR ((collaborat* OR cooperat*) NEAR/5 (doctor* OR physician* OR nurse* OR pharmacist* OR specialist* OR care OR healthcare)) OR "new role*" OR ((task* OR decision*) NEAR/2 (shift* OR reallocat* OR allocat* OR sharing OR substit*)) OR teamwork OR (team NEAR/1 (work OR approach OR member* OR training OR educat* OR interact*)) OR (( multidisciplin* OR multi-disciplin* OR interdisciplin* OR inter-disciplin*) NEAR/2 (team* OR round*)) OR ((Shift* OR liaison* OR coordinat*) NEAR/2 (care OR rore*)) OR ((change* OR extend* OR expand* OR transform*) NEAR/2 (responsib* OR skill* OR boundar* OR competenc* OR boundar*)) OR ((non-medical* OR nonmedical* OR nurse* OR pharmacist* OR "nurse practitioner*" OR "nurse specialist*" OR "specialist nurse*" OR "physician assistant*" OR "medical assistant*" OR PA) NEAR/2 (prescri*)) OR ((case OR discharge* OR nurse* OR care) NEAR/1 (manag* )) OR ((service* OR skill* OR role* OR task* OR responsib*) NEAR/2 transfer*) OR ((nurse* OR pharmacist* OR physician-assistant* OR pa OR medical-assistant* OR dentist* OR dentalassistant* OR physiotherapist* OR physical-therapist*) NEAR/1 (led OR intervention* OR managed OR run OR directed)) OR (Substitut* NEAR/2 (doctor* OR physician* OR nurse* OR pharmacist* OR specialist*)) OR ((care OR healthcare) NEAR/1 coordinat*) OR delegation OR (exten* NEAR/2 role*) OR (professional* NEAR/2 (autonom* OR boundar*)) OR (role* NEAR/5 ("nurse practitioner*" OR "nurse specialist*" OR "specialist nurse*" OR "physician assistant*" OR "medical assistant*" OR "palliative care*" OR "end of life*" OR "informal care*" OR "family care*")) OR "new role*" OR "chang* role*" OR "shared care" OR "joined consult*" OR "Patient navigat*" OR ((additional OR advanced OR new OR extended OR changed OR expanded OR supplementary OR joint OR shared OR sharing) NEAR/5 (task* OR role* OR skill* OR competenc* OR responsib*)) OR (Replace* NEAR/2 (care OR healthcare)) OR ((new OR expanded OR enlarged OR advanced) NEAR/2 (scope*-of-practice)) OR (shar* NEAR/2 decision*))) AND ((("health care" OR healthcare) NEAR/2 (personnel* OR staff* OR worker* OR workforce*) OR nurse* OR doctor* OR ((clinical OR health* OR care OR medical OR end-of-life) NEAR/2 (manpower* OR workforce* OR human-resource* OR personnel* OR professional* OR staff* OR worker* OR visitor* OR provider* OR assistant*)) OR Physician* OR general-practitioner OR doctor* OR consultant* OR nurse OR specialist* OR clinician* OR ((Advanced OR mid-level ) NEAR/2 (Pract* OR provider*)) OR physician-assistant* OR medical-assistant OR physiotherapist* OR physi*-therapist* OR occupational-therapist* OR midwif* OR midwiv* OR dentist* OR dental-staff OR pharmacist* OR pharmac*-technic* OR medical-assist* OR MD-extender* OR physician-extender* OR psychiatrist* OR psychologist* OR psychotherapist* OR Dietician* OR Dietitian* OR nutritionist* OR ((speech OR language) NEAR/2 (therapist* OR pathologist*)) OR logopaedist* OR logopedist* OR audiologist* OR ophthalmologist* OR optometrist* OR caregiver* OR carer* OR caretaker* OR (communit* NEAR/2 professional*) OR paramedic* OR gp OR gps OR ((practice* OR gp) NEAR/2 receptionist*))) AND ((((systematic*) NEAR/2 review*) OR meta-analys*)) ) NOT TI=(case report) AND DT=(article) and La=(english)

**Google scholar**

"skill mix"|"changing roles" "health care"|healthcare|nurse|doctor|medical|Physician intitle:"systematic review"|"meta analysis"

Supplement 2: Reporting checklist based on the Preferred Reporting Items for Overview of Reviews (PRIOR) guideline

| Section topic | Number | Item | Location reported |
| --- | --- | --- | --- |
| **TITLE** |  |  |  |
| Title | 1 | Identify the report as an overview of reviews | Yes - title |
| **ABSTRACT** |  |  |  |
| Abstract | 2 | Provide a comprehensive and accurate summary of the purpose, methods, and results of the overview of reviews | Yes - abstract |
| **INTRODUCTION** |  |  |  |
| Rationale | 3 | Describe the rationale for conducting the overview of reviews in the context of existing evidence | Yes – introduction |
| Objectives | 4 | Provide an explicit statement of the objective(s) or question(s) addressed by the overview of reviews | Yes – introduction, last paragraph |
| **METHODS** |  |  |  |
| Eligibility criteria | 5a | Specify the inclusion and exclusion criteria for the overview of reviews. If supplementary primary studies were included, this should be stated with a rationale. | Yes – methods, paragraph three (‘*Types of studies’).* No supplementary primary studies were included. |
|  | 5b | Specify the definition of ‘systematic review’ as used in the inclusion criteria for the overview of reviews. | Yes – included in Methods (under ‘Types of studies’). |
| Information sources | 6 | Specify all databases, registers, websites, organizations, reference lists, and other sources searched or consulted to identify systematic reviews and supplementary primary studies (if included). Specify the date when each source was last search or consulted. | Yes – methods, (‘*Electronic searches’ paragraph).* Full details of search strategy in Supplementary materials *(Supplement 1)* |
| Search strategy | 7 | Present the full search strategy strategies for all databases, registers, and websites, such that they could be reproduced. Describe any search filters and limits applied. | Yes - full details of search strategy in Supplementary materials *(Supplement 1)* |
| Selection process | 8a | Describe the methods used to decide whether a systematic review or supplementary primary study (if included) met the inclusion criteria of the overview of reviews. | Yes – methods, (‘*Selection of reviews'* paragraph) |
|  | 8b | Describe how overlap in the population, interventions, comparators, and/or outcomes of systematic reviews was identified and managed during the study selection. | Not done |
| Data collection process | 9a | Describe the methods used to collect data from the reports. | Yes – methods (‘*Data extraction and management’* paragraph*)* |
|  | 9b | If applicable, describe the methods used to identify and manage primary study overlap at the level of the comparison and outcome during data collection. For each outcome, specify the method used to illustrate and/or quantify the degree of primary study overlap across systematic reviews. | Not done, no primary studies were included |
|  | 9c | If applicable, specify the methods used to manage discrepant data across systematic reviews during data collection. | N/A |
| Data items | 10 | List and define all variables and outcomes for which data were sought. Describe any assumptions made and/or measures taken to identify and clarify missing or unclear information. | Yes – methods (‘*Data extraction and management’ and ‘Data synthesis’* paragraphs*)* |
| Risk of bias assessment | 11a | Describe methods use to *assess* risk of bias or methodological quality of the included systematic reviews. | Yes – methods (‘*Quality assessment of the reviews’* paragraph) |
|  | 11b | Describe methods use to *collect* data on (from the systematic reviews) and/or *assess* the risk of bias of the primary studies included in the systematic reviews. Provide a justification for instances where flawed, incomplete, or missing assessment are identified but not re-assessed. | Not done, data were not specifically collected from systematic reviews (other than what was reported in the publications) and primary studies were not included |
|  | 11c | Describe methods use to *assess the* risk of bias of supplemental primary studies (if included) | N/A |
| Synthesis methods | 12a | Describe the methods used to summarize or synthesize results and provide a rationale for the choice(s) | Yes – methods (‘*Data synthesis’* paragraph*)* |
|  | 12b | Describe any methods used to explore possible causes of heterogeneity among results. | Not done |
|  | 12c | Describe any sensitivity analyses conducted to assess the robustness of the synthesised results. | None, as narrative synthesize used no meta-analysis |
| Reporting bias assessment | 13 | Describe the methods used to *collect* data on (from the systematic reviews) and/or *assess* the risk of bias due to missing results in a summary or synthesis (arising from reporting biases at the levels of the systematic reviews, primary studies, and supplementary primary studies. If included) | Not done, data were not specifically collected from systematic reviews (other than what was reported in the publications) and primary studies were not included |
| Certainty assessment | 14 | Describe the methods used to *collect* data on (from the systematic reviews) and/or *assess* certainty (or confidence) in the body of evidence for an outcome | We reported the certainty assessment as reported in the systematic reviews, no primary studies were included |
| **RESULTS** |  |  |  |
| Systematic review and supplemental primary study selection | 15a | Describe the results of the search and selection process, including the number of records screened, assessed for eligibility, and included in the overview of reviews, ideally with a flow diagram | Yes – methods, Figure 1 |
|  | 15b | Provide a list of studies that might appear to meet the inclusion criteria, but were excluded, with the main reasons for exclusion. | Not included |
| Characteristics of systematic reviews and supplementary primary studies | 16 | Cite each included systematic review and supplementary primary study (if included) and present its characteristics | Yes – results, including in Tables 1-5 |
| Primary study overlap | 17 | Describe the extent of primary study overlap across the included systematic reviews. | No primary studies were included |
| Risk of bias in systematic reviews, primary studies, and supplementary primary studies | 18a | Present assessments of risk of bias or methodological quality for each included systematic review. | Yes – Results, Table 2; Supplementary materials, (*Supplement II)*. |
|  | 18b | Present assessments (collected from the systematic reviews or *assessed* anew) of the risk of bias of the primary studies included in the systematic reviews | Done as part of the quality appraisals for systematic reviews, no primary studies included |
|  | 18c | Present assessments of the risk of bias of supplementary primary studies (if included) | N/A |
| Summary or synthesis of results | 19a | For all outcomes, summarize the evidence from the systematic reviews and supplemental primary studies (if included). If meta-analysis were done, present for each the summary estimate and its precision and measures of statistical heterogeneity. If comparing groups, describe the direction of the effect. | Yes – Results, including Tables 1-5, Table 2. No meta-analysis conducted. |
|  | 19b | If meta-analysis were done, present results of all investigations of possible causes of heterogeneity. | N/A |
|  | 19c | If meta-analysis were done, present results of all sensitivity analyses conducted to assess the robustness of synthesized results. | N/A |
| Reporting biases | 20 | Present assessments (*collected* from the systematic reviews, and/or *assessed* anew) of the risk of bias due to missing primary studies, analyses, or results in a summary or synthesis (arising from reporting biases at the levels of the systematic reviews, primary studies, and supplementary primary studies, if included) for each summary or synthesis assessed) | Not done |
| Certainty of evidence | 21 | Present assessments (*collected* from the systematic reviews, and/or *assessed* anew) of certainty (or confidence) in the body of evidence for each outcome. | Not done |
| **DISCUSSION** |  |  |  |
| Discussion | 22a | Summarize the main findings, including any discrepancies in findings across the included systematic reviews and supplemental primary studies (if included). | Done for systematic reviews, no primary studies included |
|  | 22b | Provide a general interpretation of the results in the context of other evidence. | Yes, see discussion |
|  | 22c | Discuss any limitation of the evidence from systematic reviews, their primary studies, and supplemental primary studies (if included) included in the overview of reviews | Yes, see discussion, *limitations* sub-section |
|  | 22d | Discuss implications for practice, policy, and future research (both systematic reviews and primary research). Consider the relevance of the findings to the end users of the overview of reviews, e.g., healthcare providers, policymakers, patients, among others. | Yes, discussion and conclusion consider implications for future research and implementation |
| **OTHER INFORMATION** |  |  |  |
| Registration and protocol | 23a | Provide registration information for the overview of reviews, including register name and registration number, or state that the overview of reviews was not registered. | Yes – methods paragraph one (Protocol was registered in PROSPERO (Nr CRD42018090272) |
|  | 23b | Indicate where the overview of reviews protocol can be accessed, or state that a protocol was not prepared. | Yes – methods paragraph one |
|  | 23c | Describe and explain any amendments to information provided at registration or in the protocol. Indicate the stage of the overview of reviews at which amendments were made. | Yes – methods paragraph one |
| Support | 24 | Describe sources of financial or non-financial support for the overview of reviews, and the role of the funders or sponsors in the overview of reviews. | Yes |
| Competing interests | 25 | Declare any competing interests of the overview of reviews' authors. | Yes |
| Author information | 26a | Provide contact information for the corresponding author. | Yes |
|  | 26b | Describe the contributions of individual authors and identify the guarantor of the overview of reviews. | Yes |
| Availability of data and other materials | 27 | Report which of the following are available, where they can be found, and under which conditions they may be accessed: template data collection forms; data collected from included systematic reviews and supplemental primary studies; analytic code; any other materials used in the overview of reviews. | Yes – protocol and search published, template collection form, included reviews and data collected upon individual request |

Supplement 3: AMSTAR II: Quality appraisal of the systematic reviews, based on 16 items

|  | *AMSTAR II Items ** | | | | | | | | | | | | | | | | |
| --- | --- | --- | --- | --- | --- | --- | --- | --- | --- | --- | --- | --- | --- | --- | --- | --- | --- |
| Item (*see Notes)* | **Nr 1** | **2** | **3** | **4** | **5** | **6** | **7** | **8** | **9** | **10** | **11** | **12** | **13** | **14** | **15** | **16** | **Sum score** |
| Maternal and child health |  |  |  |  |  |  |  |  |  |  |  |  |  |  |  |  |  |
| Kroll-Desrosiers et al. (2016) | 0 | 1 | 0 | 1 | 0 | 0 | 0 | 1 | 0 | 0 | 1 | 1 | 0 | 0 | 1 | 2 | 8 |
| Lewin et. al. (2010) | 2 | 2 | 0 | 2 | 2 | 2 | 2 | 2 | 2 | 0 | 2 | 2 | 2 | 2 | 0 | 0 | 24 |
| Farris et al. (2010) | 0 | 0 | 0 | 1 | 2 | 0 | 0 | 1 | 0 | 0 | 1 | 1 | 0 | 0 | 1 | 2 | 9 |
| Khan-Neelofur (1998) | 2 | 1 | 0 | 1 | 0 | 2 | 0 | 2 | 2 | 0 | 1 | 1 | 0 | 2 | 1 | 0 | 15 |
| Abbott & Elliott (2017) | 2 | 1 | 0 | 1 | 0 | 2 | 0 | 1 | 0 | 0 | 1 | 1 | 0 | 0 | 1 | 2 | 12 |
| Issel et. al (2011) | 0 | 0 | 0 | 2 | 2 | 2 | 0 | 1 | 2 | 0 | 1 | 1 | 0 | 0 | 1 | 2 | 14 |
| Dalziel and Segal (2012) | 2 | 1 | 0 | 2 | 2 | 2 | 0 | 2 | 2 | 0 | 1 | 1 | 2 | 2 | 1 | 2 | 22 |
| Screening |  |  |  |  |  |  |  |  |  |  |  |  |  |  |  |  |  |
| Wells et al. (2011) | 0 | 1 | 0 | 0 | 0 | 2 | 0 | 1 | 1 | 0 | 2 | 2 | 2 | 2 | 0 | 2 | 15 |
| Jopseph et.al (2015) | 0 | 0 | 0 | 1 | 0 | 0 | 0 | 1 | 0 | 0 | 1 | 1 | 0 | 0 | 1 | 2 | 7 |
| Loescher, Harris, & Curiel-Lewandrowski (2011) | 0 | 1 | 0 | 1 | 0 | 0 | 0 | 0 | 1 | 0 | 1 | 1 | 0 | 2 | 1 | 0 | 8 |
| Kim et al. (2016) | 0 | 0 | 0 | 1 | 2 | 0 | 0 | 1 | 1 | 0 | 1 | 1 | 0 | 0 | 1 | 0 | 8 |
| Heuvelings et al (2018) | 2 | 1 | 0 | 2 | 2 | 2 | 0 | 1 | 2 | 0 | 1 | 1 | 2 | 0 | 1 | 2 | 19 |
| Vaccinations |  |  |  |  |  |  |  |  |  |  |  |  |  |  |  |  |  |
| Lewin et. al. (2010) | 2 | 2 | 0 | 2 | 2 | 2 | 2 | 2 | 2 | 0 | 2 | 2 | 2 | 2 | 0 | 0 | 24 |
| Spinks et al (2020) | 0 | 0 | 0 | 1 | 2 | 0 | 0 | 0 | 0 | 0 | 1 | 1 | 2 | 0 | 1 | 2 | 10 |
| Lifestyle (SNAPW) |  |  |  |  |  |  |  |  |  |  |  |  |  |  |  |  |  |
| Saba et al. (2014) | 2 | 2 | 0 | 1 | 2 | 2 | 0 | 2 | 2 | 0 | 2 | 2 | 2 | 2 | 0 | 2 | 23 |
| Bhattarai et al. (2013) | 2 | 0 | 0 | 1 | 0 | 2 | 0 | 1 | 1 | 0 | 2 | 0 | 0 | 0 | 2 | 2 | 13 |
| Ball et al. (2015) | 0 | 0 | 0 | 1 | 2 | 2 | 0 | 2 | 2 | 0 | 1 | 1 | 0 | 0 | 1 | 2 | 14 |
| Mitchell et al. (2017) | 2 | 2 | 2 | 1 | 2 | 2 | 0 | 2 | 2 | 0 | 1 | 1 | 2 | 2 | 1 | 2 | 24 |
| Thompson et al. (2003) | 2 | 0 | 0 | 2 | 2 | 2 | 2 | 2 | 1 | 0 | 2 | 2 | 2 | 2 | 2 | 0 | 23 |
| Orrow et al. (2012) | 2 | 0 | 0 | 1 | 2 | 2 | 0 | 2 | 2 | 0 | 2 | 2 | 0 | 2 | 2 | 2 | 21 |
| Kunstler et al. (2018) | 2 | 2 | 0 | 1 | 2 | 2 | 0 | 2 | 2 | 0 | 2 | 2 | 2 | 2 | 0 | 2 | 23 |
| Schroeder et al. (2016) | 0 | 1 | 0 | 1 | 0 | 0 | 0 | 1 | 1 | 0 | 2 | 2 | 0 | 2 | 2 | 0 | 12 |
| Sargent et al. (2012) | 0 | 0 | 0 | 1 | 2 | 0 | 0 | 2 | 2 | 0 | 1 | 1 | 2 | 0 | 1 | 2 | 14 |
| Petit Francis et al. (2017) | 0 | 1 | 0 | 1 | 2 | 2 | 0 | 2 | 1 | 0 | 1 | 1 | 2 | 0 | 1 | 2 | 16 |
| Van Dillen & Hiddink (2014) | 0 | 0 | 0 | 0 | 0 | 0 | 0 | 2 | 0 | 0 | 1 | 1 | 0 | 0 | 1 | 2 | 7 |
| Flodgren et al. (2017) | 2 | 2 | 0 | 1 | 2 | 2 | 2 | 2 | 2 | 2 | 2 | 2 | 2 | 2 | 2 | 2 | 29 |
| Martínez-González et al. (2015) | 2 | 1 | 2 | 2 | 2 | 2 | 2 | 2 | 2 | 2 | 1 | 0 | 2 | 2 | 1 | 2 | 27 |
| Brown et al. (2016) | 2 | 2 | 2 | 2 | 2 | 2 | 0 | 1 | 2 | 1 | 2 | 0 | 0 | 2 | 2 | 2 | 24 |
| Dennis et al. (2012) | 0 | 1 | 0 | 1 | 2 | 2 | 0 | 2 | 1 | 0 | 1 | 1 | 0 | 0 | 1 | 2 | 14 |
| Pennington et al. (2013) | 2 | 1 | 0 | 1 | 2 | 2 | 0 | 1 | 1 | 1 | 1 | 1 | 0 | 0 | 1 | 2 | 16 |
| Tapsell et al. (2016) | 0 | 0 | 0 | 1 | 0 | 0 | 0 | 1 | 0 | 0 | 1 | 1 | 0 | 0 | 1 | 2 | 7 |
| Frerichs et al. (2012) | 0 | 0 | 0 | 1 | 2 | 2 | 0 | 1 | 2 | 0 | 1 | 1 | 0 | 2 | 1 | 2 | 15 |

Source: AMSTAR II (39).

Notes: AMSTAR: Assessment of Multiple Systematic Reviews, *AMSTAR II Items: Item 1: Did the research questions and inclusion criteria include the PICO Elements (Population, Intervention, Comparator, Outcomes)? Yes(=2): all 4 PICOs and (optionally) timeframe of follow-up mentioned. No (=0): not all 4 PICO mentioned;

2: Was the methodology established prior to the conduct and did the report justify any signifcant deviation from the protocol? For Partial Yes (=1): protocol included review questions, search strategy, inclusion/exclusion criteria, risk of bias. For Yes (=2): additionally, the registered protocol should entail: meta analysis/synthesis plan, investigating in causes of heterogeneity, justification for deviation from protocol. No (=0).

3. Is there an explanation of the selection of study design included? For Yes (=2) at least ONE of the following: Explanation for including only RCTs, explanation for only including NRSI, explaination for including both RCTs and NRSIs. No(=0).

4. Was a comprehensive literature search applied? For partial Yes (=1) all of: searched at least 2 databases, provided key word and search strategy, justified publication restrictions. For Yes (=2) additionally: searched the reference lists, searched trial/study registries, consulted experts in the field, searched for grey literature, conducted search within 24 months of completion of the review. No (=0).

5. Did the authors perform study selection in duplicate? For Yes (=2) one of: at least two reviewers independently agreed on selection of eligible studies and achieved consensus, OR two reviewers selected a sample of eligible studies and achieved good agreemend (at least 80 %) with the remainder selected by one reviewer. No (=0).

6. Did the authors perform data extraction in duplicate? For Yes (=2) at least ONE: at least two reviewers achieved consensus on which data to extract from included studies, OR two reviewers extracted data from a sample of eligible studies and achieved good agreement ( at least 80%) with the remainder extracted by one reviewer. No (=0).

7. Was a list of excluded studies provided with justification of exlusion? For Partial Yes (=1): provided a list of all potentially relevant studies that were read in full text form but excluded from the review. For Yes (=2) additionally: justified the exclusion from the review of each potentially relevant study.

8. Did the authors describe included studies in adequate detail? For Partial Yes (=1): described populations, interventions, comparators, outcomes, research designs. For Yes (=2) additionally: described population, intervention (incl. Doses), comparator in DETAIL, described setting and time frame for follow-up. No (=0).

9. Did the authors use a satisfactory technique for assessing risk of bias in individual studies (RCT/NRSI) that were included? Please refer to the AMSTAR 2 Checklist for detailed information. Yes (=2). Partial Yes (=1). No (=0).

10. Did the authors report on the source of funding for the studies included in the review? For Yes (=2): Must have reported on the sources of funding for individual studies included. Note: reporting that the reviewers looked for it but it was not reported by study authors also qualifies.

11. If meta-analysis was performed, did the authors use appropriate methods for statistical combination of results? Please refer to the AMSTAR 2 Checklist for detailed information. Yes (=2). No (=0). No meta analysis conducted (=1).

12. If meta-analysis was performed, did the authors assess the potential impact of risk of bias (RoB) in individual studies on the results of the meta-analysis or evidence synthesis? For Yes (=2): included only low risk of bias RCTS OR if the pooled estimate was based on RCTs and NRSI at variable RoB, the authors performed analyses to investigate possible impact if RoB on summary estimate of effect. No (=0). No meta analysis conducted (=1).

13. Did the authors account for RoB in the studies when interpreting and discussing the results? For Yes (=2): included only low risk of bias RCTs OR if RCTs with moderate or high RoB or NRSI were included, the review provides discussion of likely impact of RoB on results. No (=0).

14. Did the authors provide a satisfactory explaination for and discussion of any heterogeinity observed in the results? For Yes (=2): There was no significant heterogeinity in the results OR if heterogeinity was present the authors performed an investigation of its sources and discussed the impact on results. No (=0).

15. If quantitative synthesis was performed, did authors carry out investigation into publication bias (small study bias) and discuss ist possible impact on results? For Yes (=2): performed graphical or statistical tests for publication bias and discussed likelihood and magnitude of impact. No (=0). No meta-analysis was conducted (=1).

16. Did the authors report on any potential source of conflict? For Yes (=2): the authors reported no competing interests OR the authors described their funding sources and how they managed potential conflicts of interest. No (=0).
